# A Molecular Phylogeny for Yponomeutoidea (Insecta, Lepidoptera, Ditrysia) and Its Implications for Classification, Biogeography and the Evolution of Host Plant Use

**DOI:** 10.1371/journal.pone.0055066

**Published:** 2013-01-31

**Authors:** Jae-Cheon Sohn, Jerome C. Regier, Charles Mitter, Donald Davis, Jean-François Landry, Andreas Zwick, Michael P. Cummings

**Affiliations:** 1 Department of Entomology, University of Maryland, College Park, Maryland, United States of America; 2 Department of Entomology, National Museum of Natural History, Smithsonian Institution, Washington DC, United States of America; 3 Agriculture and Agri-Food Canada, Eastern Cereal and Oilseed Research Centre, C.E.F., Ottawa, Canada; 4 Department of Entomology, State Museum of Natural History, Stuttgart, Germany; 5 Laboratory of Molecular Evolution, Center for Bioinformatics and Computational Biology, University of Maryland, College Park, Maryland, United States of America; Centre National de la Recherche Scientifique, France

## Abstract

**Background:**

Yponomeutoidea, one of the early-diverging lineages of ditrysian Lepidoptera, comprise about 1,800 species worldwide, including notable pests and insect-plant interaction models. Yponomeutoids were one of the earliest lepidopteran clades to evolve external feeding and to extensively colonize herbaceous angiosperms. Despite the group’s economic importance, and its value for tracing early lepidopteran evolution, the biodiversity and phylogeny of Yponomeutoidea have been relatively little studied.

**Methodology/Principal Findings:**

Eight nuclear genes (8 kb) were initially sequenced for 86 putative yponomeutoid species, spanning all previously recognized suprageneric groups, and 53 outgroups representing 22 families and 12 superfamilies. Eleven to 19 additional genes, yielding a total of 14.8 to 18.9 kb, were then sampled for a subset of taxa, including 28 yponomeutoids and 43 outgroups. Maximum likelihood analyses were conducted on data sets differing in numbers of genes, matrix completeness, inclusion/weighting of synonymous substitutions, and inclusion/exclusion of “rogue” taxa. Monophyly for Yponomeutoidea was supported very strongly when the 18 “rogue” taxa were excluded, and moderately otherwise. Results from different analyses are highly congruent and relationships within Yponomeutoidea are well supported overall. There is strong support overall for monophyly of families previously recognized on morphological grounds, including Yponomeutidae, Ypsolophidae, Plutellidae, Glyphipterigidae, Argyresthiidae, Attevidae, Praydidae, Heliodinidae, and Bedelliidae. We also assign family rank to Scythropiinae (Scythropiidae **stat. rev.**), which in our trees are strongly grouped with Bedelliidae, in contrast to all previous proposals. We present a working hypothesis of among-family relationships, and an informal higher classification. Host plant family associations of yponomeutoid subfamilies and families are non-random, but show no trends suggesting parallel phylogenesis. Our analyses suggest that previous characterizations of yponomeutoids as predominantly Holarctic were based on insufficient sampling.

**Conclusions/Significance:**

We provide the first robust molecular phylogeny for Yponomeutoidea, together with a revised classification and new insights into their life history evolution and biogeography.

## Introduction

The Yponomeutoidea constitute one of the early radiations in the so-called ditrysian Lepidoptera, the advanced clade that contains the great majority of lepidopteran species. Yponomeutoids include about 1,800 species worldwide, known heretofore mainly from temperate regions [1], [2]. Yponomeutoidea are especially important for tracing the early evolution of Lepidoptera-plant interactions because they are one of the earliest groups to evolve external feeding [3] and to extensively colonize herbs as well as shrubs and trees [4]. In the modern fauna, those two traits are especially common in the highly diverse lineages of advanced moths, for whose success they may be in part responsible. Some yponomeutoid groups, especially *Yponomeuta*, have served as model systems in studying how insect-plant interactions affect speciation [5]. Yponomeutoidea also include a number of notable pest species. For example, the diamondback moth (*Plutella xylostella*: Plutellidae) is regarded as the most destructive insect pest of cruciferous vegetables, annually causing about a billion US dollars in economic loss [6]. Another notorious pest, the leek moth (*Acrolepiopsis assectella*: Glyphipterigidae), has caused damage to upwards of 70% of leeks and 40–50% of onions in some regions of Europe [7]. Communal larvae of some species sometimes extensively damage local vegetation or even broader landscapes. The small ermine moths (*Yponomeuta* spp.) cause complete defoliation of some trees in northern Europe (e.g. [8], [9]) and the U.S. (e.g. the introduced *Y. malinellus* [10]).

**Figure 1:**
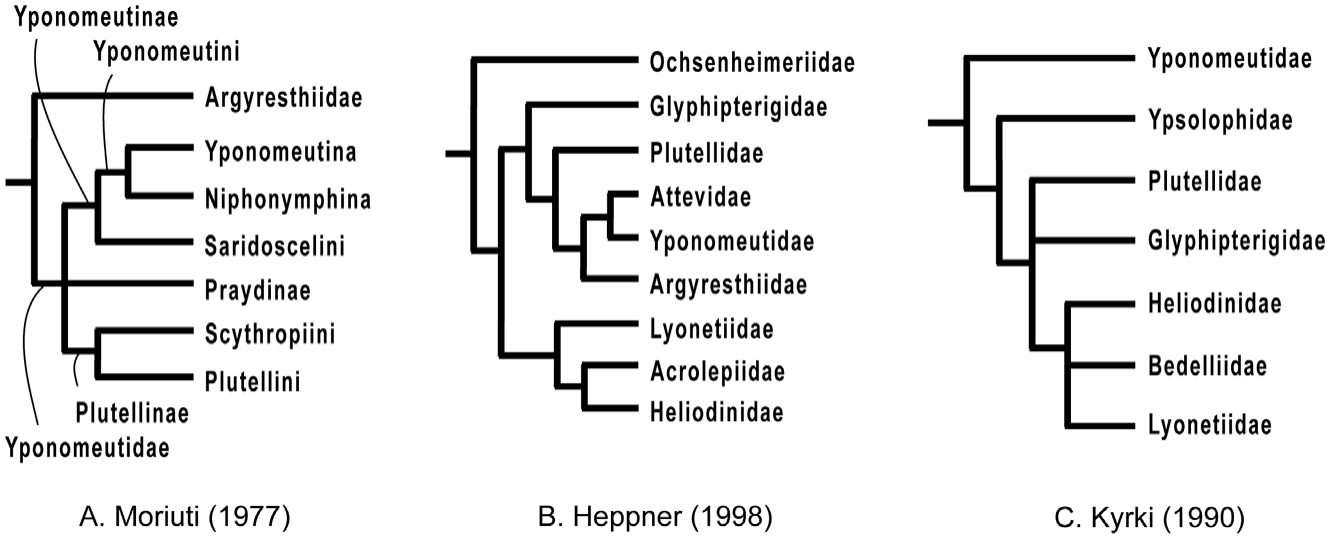
Previous hypotheses of phylogenetic relationships in Yponomeutoidea. A. Moriuti (1977), B. Heppner (1998), C. Kyrki (1990). All figures are redrawn with nomenclature following the original.

Despite their value for tracing the early evolution of Lepidoptera and their importance as pests, the Yponomeutoidea have received relatively little attention from systematists, and their biodiversity remains poorly understood. Especially problematic is the lack of a robust phylogeny, including a synapomorphy-based definition for the superfamily itself. Until the early 20^th^ century, the taxa currently placed in Yponomeutoidea comprised scattered suprageneric groups of Tineina or Tineae, two collective microlepidopteran group names no longer in use (e.g. [11], [12], [13], [14]), or Tineidae (e.g. [15], [16]). Although Stephens [17] had already distinguished them from other microlepidopteran groups, it was Fracker [18] who first erected a superfamily for Yponomeutoidea. However, as it lacked unambiguously defining characters, the group remained highly heterogeneous and included many genera that now belong to other superfamilies. A succession of subsequent authors advanced increasingly restrictive re-definitions of Yponomeutoidea (e.g. [14], [19], [20], [21], [22], [23], [24]), but failed to achieve a stable classification because they lacked explicit analyses of phylogenetic relationships (Table 1). Kyrki [25], [26], in the first cladistic study, significantly modernized the classification of Yponomeutoidea, in which he included only seven families: Yponomeutidae, Ypsolophidae, Plutellidae, Glyphipterigidae, Heliodinidae, Bedelliidae and Lyonetiidae. However, the lack of robustness of Kyrki’s phylogeny hindered acceptance of his classification, leaving other hypotheses, such as those of Moriuti [27] and Heppner [1], still in contention (Fig. 1). Disagreements on the phylogeny of Yponomeutoidea, in turn, have helped to obscure inter-relationships of the basal lepidopteran groups and hindered testing of evolutionary hypotheses bearing on them.

**Table 1:** Previous classifications of Yponomeutoidea.

**Common (1970)**	**Moriuti (1977)**	**Heppner (1998)**	**Kyrki (1990)**	**van Nieukerken et al. (2011)**
**Yponomeutidae**	**Yponomeutidae**	**Yponomeutidae**	**Yponomeutidae**	**Yponomeutidae**
Plutellinae	Yponomeutinae	Yponomeutinae	Yponomeutinae	Yponomeutinae
Yponomeutinae	Yponomeutini	Saridoscelinae	Saridoscelinae	Saridoscelinae
Amphitherinae	Yponomeutina	Cedestinae	Scythropiinae	Scythropiinae
Argyresthinae	Niphonymphina	**Attevidae**	Attevinae	**Attevidae**
**Glyphipterigidae**	Saridoscelini	**Argyresthiidae**	Praydinae	**Praydidae**
**Heliodinidae**	Praydinae	**Plutellidae**	Argyresthiinae	**Argyresthiidae**
**Aegeriidae**	Plutellinae	Ypsolophinae	**Plutellidae**	**Plutellidae**
**Douglasiidae**	Scythropiini	Plutellinae	Plutellinae	**Ypsolophidae**
**Epermeniidae**	Plutellini	Scythropiinae	Acrolepiinae	Ypsolophinae
	**Argyresthiidae**	Praydinae	**Ypsolophidae**	Ochsenheimeriinae
		**Acrolepiidae**	Ypsolophinae	**Glyphipterigidae**
		**Ochsenheimeriidae**	Ochsenheimeriinae	Acrolepiinae
		**Glyphipterigidae**	**Glyphipterigidae**	Orthoteliinae
		Orthoteliinae	Orthoteliinae	Glyphipteriginae
		Glyphipteriginae	Glyphipteriginae	**Heliodinidae**
		**Heliodinidae**	**Heliodinidae**	**Lyonetiidae**
		**Lyonetiidae**	**Lyonetiidae**	Cemiostominae
		Cemiostominae	Cemiostominae	Lyonetiinae
		Lyonetiinae	Lyonetiinae	**Bedelliidae**
		Bedelliinae	**Bedelliidae**	

Nomenclature follows the original. Families are indicated in bold.

Recent molecular studies of higher phylogeny in Lepidoptera have begun to clarify the phylogenetic position, definition and internal relationships of Yponomeutoidea [28], [29], [30]. The results of Mutanen et al. [29], who included 23 yponomeutoids in an analysis of 350 lepidopterans sequenced for 8 genes (6.3 kb), were the basis for the revised 10-family classification (Table 1) of van Nieukerken et al. [2]. Here, in the first molecular study aimed specifically at Yponomeutoidea, we greatly expand previous taxon and gene sampling, providing the most comprehensive examination and robust hypothesis to date of phylogeny in this superfamily. We compare our results to all previous classification systems, then trace evolutionary trends in yponomeutoid host associations and biogeography on the new phylogeny.

## Materials and Methods

### Taxon Sampling

A total of 86 species currently assigned to Yponomeutoidea were included in our analyses. These represent all 17 suprageneric groups recognized by Kyrki [25], and all 10 families recognized by van Nieukerken et al. [2] as well as all subfamilies and tribes therein. The sample collectively spans nearly all zoogeographical regions, including 37 species from the Palearctic, 21 from the Neotropics, 17 from the Nearctic, seven from the Australian region, two from the Oriental region, and two from the Ethiopian region. All yponomeutoid genera for which material could be obtained were included, each represented by a single species except that two or more species were sampled for several broadly distributed, species-rich genera.

The definition of Yponomeutoidea has been considered controversial [31]. For this reason, our putative outgroups, totaling 53 species belonging to 22 families in 12 superfamilies of ditrysian Lepidoptera (see Supplement S1), included all superfamilies that were historically associated with Yponomeutoidea or at least contain genera that were once placed within Yponomeutoidea. Among these are Choreutoidea, Copromorphoidea, Epermenioidea, Galacticoidea, Gelechioidea, Schreckensteinoidea, Urodoidea, and Zygaenoidea. Inclusion of these taxa provides an additional test of the monophyly of Yponomeutoidea in the restricted modern sense. We also included two superfamilies, Tortricoidea and Pterophoroidea, which have never been considered close to yponomeutoids. In contrast to all previous hypotheses, recent molecular studies [28], [29], [30] have strongly supported Gracillarioidea as the closest relatives to Yponomeutoidea sensu Kyrki [25], [26]. For this reason we sampled gracillarioids especially densely, taking exemplars from most of the known families and subfamilies. We included comparably dense sampling of Tineoidea, which have long been considered, now with increasing molecular evidence ([29] and J. Regier et al., unpublished results), to contain the earliest-branching lineages within the Ditrysia [32]. Finally, to root the entire tree, we added a representative of Tischeriidae, long regarded, also with increasing molecular evidence ([29] and J. Regier et al., unpublished results), to be among the closest relatives to Ditrysia.

### Specimen Preparation and Identification

The specimens for this study, obtained by our own collecting as well as from collaborators around the world (see Acknowledgments), are stored in 100% ethanol at −80°C as part of the ATOLep frozen tissue collection at the University of Maryland, College Park, USA (details at http://www.leptree.net/collection). For extraction of nucleic acids we used the legs, head and thorax, or the entire body (always excluding the wings), depending on the size of the specimen. As vouchers we preserved both wings and abdomen for large or medium-sized moths, and wings only for very small ones. Wing voucher images for most of our specimens are available at the Leptree website (http://www.leptree.net/voucher_image_list). Partial COI sequences corresponding to DNA ‘barcodes’ were generated for each specimen either by the authors or as part of the All-Leps Barcode of Life project (http://www.lepbarcoding.org). Using these sequences, we performed an independent check of the primary identifications of all specimens by searching for matching barcode sequences in the BOLD (Barcode of Life Data system, http://www.boldsystems.org).

### Gene Sampling

The sequences initially sampled for this study consisted of eight nuclear genes (Supplment S1), totaling 8,096 bp, for nearly all ingroup taxa (83/86 = 96.5%) and all outgroup taxa. These eight are a subset of the 26 genes sequenced in a study of ditrysian phylogeny by Cho et al. [30], 25 of which were also analyzed in Bombycoidea by Zwick et al. [33]. The eight gene subset was chosen on the basis of its relatively high amplification success rates and phylogenetic utility. The eight genes are: *Gelsolin* (603 bp), *histidyl tRNA synthetase* (447 bp), *AMP deaminase* (768 bp), *glucose phosphate dehydrogenase* (621 bp), *Acetyl-coA carboxylase* (501 bp), *CAD* (2,929 bp), *DDC* (1,281 bp) and *enolase* (1,135 bp). Three species (*Argyresthia austerella*, *Digitivalva hemiglypha*, and *Prays atomocella*), each with close relatives in the eight gene data set, were sequenced for only the five genes (6.6 kb) studied in Ditrysia by Regier et al. [28], namely, *CAD*, *DDC*, *enolase*, *period*, and *wingless* (Figure S1).

Because the initial 8-gene analyses yielded little strong support for deeper nodes, we subsequently added 11–19 more nuclear genes (totaling up to 27 genes and 19,386 bp) for a taxon subset consisting of 28 ingroups and 43 outgroups (Figure S1), amounting to 51% of the total of 139 taxa. The 27 genes include the 26 used by Cho et al. [30], plus one additional gene, *α-spectrin*. All 27 are included in the set of 68 genes studied by Regier et al. [34] across the arthropods. The great majority of taxa (54/65) for which more than eight genes were assayed were sequenced for just the 19 gene set that has recently proven useful in resolving relationships in other superfamilies, including Gracillarioidea [35], Tortricoidea [36] and Pyraloidea [37]. These same studies have also shown that augmentation of the initial gene sample in only a subset of taxa, following Cho et al. [30], is an effective and cost-efficient means for obtaining stronger support at deeper nodes. Partial gene augmentation introduces blocks of nonrandomly missing data that could have adverse effects on phylogeny estimation [38], [39]. To test this possibility, we compared the results from the 8+19 gene, deliberately incomplete matrix to those from a 4-gene data set (*glucose phosphate dehydrogenase*, *CAD*, *DDC* and *enolase*) that exhibit a relatively low percentage of missing data (21.5%) among our 139 taxa, due to inadvertent failures of amplification or sequencing.

### Gene Extraction, Sequencing and Alignment

A detailed protocol of all laboratory procedures is provided by Regier et al. [34]. Further descriptions, including gene amplification strategies, PCR primer sequences, sequence assembly and alignment methods, can be found in Regier [40] and Regier et al. [28], [41]. To summarize, total RNAs were extracted from an excised tissue using the SV Total RNA Isolation System (Promega Co.). The targeted regions of the mRNAs were amplified using Reverse Transcriptase (RT)-PCR, yielding cDNA. Nested PCR for further purification and/or M13 re-amplification for increasing volume were attempted as necessary. Purified amplicons were sequenced on a 3730 DNA Analyzer (Applied Biosystems) at the Center for Biosystems Research at the University of Maryland, College Park. The resulting ABI files and contigs were checked for error manually and then edited and assembled using Geneious Pro 5.3.4 (Biomatters Ltd.). The data were rechecked for error by inspection of the genetic distances among them determined in PAUP* 4.0b8 [42]. The final sequences for each gene were aligned using the “Translation Align” option in Geneious. The final alignments were concatenated with Geneious, separately for the 8-gene and 8–27 gene analyses, and the combined data sets were visually checked. Regions of uncertain alignment, totaling 1,509 characters, were masked and excluded from subsequent analyses. GenBank accession numbers and the percentage sequence completeness for each gene in each taxon are given in Figure S1.

### Character Partition and Data Set Design

It is well known that rates of sequence evolution vary among codon positions, reflecting in part different ratios of synonymous versus nonsynonymous substitutions [43], [44]. Previous empirical studies (e.g. [28], [30], [34]) have shown that partitioning data to reflect this variation, or eliminating synonymous change entirely, can reduce or eliminate phylogenetic error due to among-lineage compositional heterogeneity, but at the cost of discarding potentially informative synonymous signal. To gauge the potential effects of differing evolutionary properties between synonymous and non-synonymous substitution on phylogeny inference, we carried out separate analyses using a variety of character coding and/or data partition schemes. These analyses are: (a) “nt123”, i.e., all codon positions included and unpartitioned; (b) “degen1” [45], [46], i.e., all synonymous differences degenerated, leaving only non-synonymous differences among taxa; (c) “nt123 partitioned” [28], i.e., all codon positions partitioned into mostly non-synonymously evolving (“noLRall1+nt2”) versus mostly synonymously- evolving ones (“LRall1+nt3”); and, (d) “codon” analysis [47], [48], in which the character states are codons and synonymous and nonsynonymous changes are modeled separately. For the codon analyses (only), a 91 taxon set including only Yponomeutoidea and Gracillarioidea was used, rather than the full 139 taxon data set, to reduce the computational burden. Increased numbers of discrete rate categories in the gamma-distributed rate heterogeneity distribution (‘numratecats’ in the GARLI configuration) can also dramatically increase computational time. To avoid this problem, we used trial runs to estimate a minimum number of categories beyond which further increase yields no significant improvement in tree likelihood scores. We determined this number to be three categories. As a third approach to accommodating differences between synonymous and non-synonymous change, we also partitioned the data into first plus second codon positions (“nt12”, Figure S3) versus third codon positions (“nt3”, Figure S4).

### Phylogenetic Analyses

The best substitution model for each data set was determined using jModelTest [49], which in nearly all cases selected GTR+Г+I, i.e., the General-Time-Reversible model with among-site rate variation accomodated using a gamma distribution plus separate estimation of a proportion of invariable sites. Phylogenetic analyses were conducted with maximum likelihood (ML) methods as implemented in GARLI 2.0 [50], which includes partitioned models. Default settings of the program were used, except that starting tree topology was specified as random; the frequencies with which to log the best score (‘logevery’) and to save the best tree to file (‘saveevery’) were set to 100,000 and 100,000 respectively; and, the number of generations without topology improvement required for termination (‘genthreshfortopoterm’) was set to 5,000. The best tree from 150 independent search replicates was saved, and visualized using FigTree v1.3.1 [51]. To evaluate the robustness of the resulting trees, bootstrap (BP) values were calculated from 1000 pseudoreplicates, each based on 15 heuristic search replicates except that only a single heuristic search replicate was carried out for each pseudoreplicate in the single-gene bootstrap analyses. Because these analyses are so computation-intensive, they were carried out by Grid parallel computing [52], using the Lattice Project [53], [54]. For purposes of discussion, we will refer to BP values of 70–79% as “moderate”, 80–89% as “strong”, and ≥90% as “very strong” support. These conventions, also adopted in previous studies (e.g. [30], [35]), are arbitrary and hence serve heuristic purposes only.

### Rogue Taxon Analyses

Despite the addition of 11–19 genes to the initial 8-gene data set, some deeper nodes in even our best-supported trees have low bootstrap values. One possible cause of low support is the sensitivity of bootstrap values to taxa of unstable placement [55], termed “rogues” by Wilkinson [56]. Multiple approaches have been suggested for detecting and removing the effects of rogue taxa (reviewed in [57]). We investigated the potential contribution of rogue taxa (Table 2) to low bootstrap values in our data set using the RogueNaRok (RNR) approach of Aberer et al. ([58]; a pun on Ragnarök, the judgement of the gods in Norse mythology). The key feature of RNR is a new optimality criterion for rogue taxon removal, the “Relative Bipartition Information Criterion” (RBIC) [57], [59]. The RBIC strikes a balance between improving per-node support in the reduced bootstrap consensus tree (with rogues deleted) and retaining total information by minimizing the loss of bipartitions in the bootstrap consensus tree that results from such deletions. Aberer and Stamatakis [59] compared multiple heuristic approaches to maximizing the RBIC. The best results came from their single-taxon algorithm (STA), which begins by removing taxa one at a time to find the taxon (if any) whose deletion most improves the RBIC. After that taxon is removed, one removes each remaining taxon again, to find the next most “roguish” taxon. The process is repeated until the optimality score stops improving. The RogueNaRok algorithm is a fast generalization of the STA, which allows for “deletion sets” – groups of taxa deleted simultaneously – of varying sizes.

**Table 2:** Rogue taxa identified by the RogueNaRok (RNR) analyses, listed in the order in which they were identified and removed.

Rogue taxon set^*^	Rogue taxon	Codename	SC^**^(%)	Raw Improvement^***^	RBIC
A	*Copromorpha* sp.	Cmpa	12	0.906667	0.767598
	***Xyrosaris lichneuta***	Xlic	29.2	0.74	0.773039
	*Cycloplasis panicifoliella*	Cpan	26.2	0.666667	0.777941
	*Hybroma servulella*	Hybs	67.0	0.58	0.782206
	*Epermenia sinjovi*	Esji	30.6	0.26	0.784118
	*Philonome clemensella*	Pmsa	26.7	0.246667	0.785931
	*Opogona thiadelia*	Othi	64.1	0.113333	0.786765
	*Emmelina monodactyla*	Emon	86.9	0.093333	0.787451
	*Klimeschia transversella*	Ktr	66.4	0.906667	0.794118
	*Hemerophila felis*	Hfel	90.8	0.186667	0.79549
	*Nemapogon cloacella*	Nclo	55.1	0.013333	0.795588
B	*Narycia duplicella*	Nard	34.1	0.373333	0.867413
	*Euclemensia bassettella*	Cole	81.6	0.146667	0.868587
	*Bucculatrix* sp.	Bucc	56.9	0.033333	0.868853
C	*Homadaula anisocentra*	Hani	64.7	0.82	0.870656
	“*Wockia*” sp.	MX60	19.1	0.2	0.879016
D	***Perileucoptera coffeella***	Leuco	43.2	0.12	0.874545
	***Swammerdamia glaucella***	Swgl	33.7	0.046667	0.875076

The RBIC (relative bipartition information content) for the reduced consensus tree, after pruning all taxa up to and including any given rogue taxon, is shown in the last column. Ingroup rogue taxa are shown in bold. * Rogue taxon sets = rogue taxa identified on each successive one-at-a-time pass through the taxa. Each such pass, after the first pass, starts from a reduced taxon set from which all previously-identified rogues have been removed. Following the removal of rogue taxon sets A–C, no further rogue taxa could be identified in the entire data set. Rogue taxon set D was identified in an independent analysis of just Yponomeutoidea+Gracillarioidea, excluding other outgroups. A: 139 taxa x 8–27 genes. Initial score = 0.760931, # of partitions in reduced consensus tree = 973. B: 128 taxa (11 rogue taxa deleted from A). Initial score = 0.864427, # of partitions = 443. C: 125 taxa (3 rogue taxa deleted from B). Initial score = 0.870656, # of partitions = 337. D: 91 taxa (Yponomeutoidea+Gracillarioidea). Initial score = 0.873182, # of partitions = 272. ** SC (sequence data completeness) = (# of nucleotides actually sequenced/total # of targeted nucleotides) x 100. ***Raw Improvement: the improvement in support (sum of all bootstrap values) for the reduced consensus tree, if the taxon in question is pruned AND all previously identified rogue taxa are also pruned.

To identify rogue taxa, we used the on-line version of RogueNaRok (RNR) at http://193.197.73.70∶8080/rnr/roguenarok, which is built on RAxML [60]. Bootstrap files were first generated and submitted to RNR, which identified possible rogue taxa (i.e. ones whose removal increases the RBIC). The reduced data set was then analyzed with RAxML, and the bootstrap outputs again submitted to RNR. This procedure was repeated until RNR no longer identified any additional rogues. Finally, the putatively rogue-free data sets were subjected to bootstrap analyses using GARLI, to make them directly comparable to the original analyses. This procedure was carried out only for the nt123, 8–27 gene data set, which gave the highest initial bootstrap support overall. In our initial RNR analyses, most of the rogue taxa detected were among the more distant outgroups. This result might stem from increased uncertainty in position due to lower sampling density among these taxa, and might in turn impede detection of more subtle rogue taxon effects within the ingroup, which is what we are most interested in. To circumvent this possibility, we also conducted separate RNR analyses on data sets containing Yponomeutoidea (86 taxa) and Gracillarioidea (11 taxa) only.

### Significance Tests of Discord with Previous Hypotheses

Our results appear to contradict a number of prior hypotheses about phylogenetic relationships in Yponomeutoidea, including several depicted in Figure 1. We used the Approximately Unbiased (AU) test of Shimodaira [61] to determine whether our data significantly reject those previous hypotheses, against the alternative that the discrepancy can be explained by sampling error in the sequence data. The test determines whether the best tree possible under the constraint of monophyly, no matter what its topology may be otherwise, is a significantly worse fit to the data than the best tree without that constraint. Table 3 lists the 12 groups tested for significance of non-monophyly. For each combination of one character set and one apparently non-monophlyetic previous grouping, we performed a GARLI analysis consisting of 150 replicate tree searches, under the constraint of monophyly for the group in question. The constrained tree was then compared to the previously-obtained unconstrained tree. The site likelihoods of the best constrained and unconstrained trees were then estimated with PAUP* [42], and the trees and site likelihoods for all comparisons combined into a single input file for the CONSEL 0.20 package [62], [63] with which the Approximately Unbiased test was conducted.

**Table 3:** Results of Approximately Unbiased (AU) tests for significance of rejection of 12 previous phylogenetic hypotheses.

#	Constraint group	Source	nt123 (*p*)	degen1 (*p*)
1	Yponomeutoidea *sensu* Kyrki (Fig. 1)	Kyrki (1990)	**0.001**	**<0.001**
2	Yponomeutoidea *sensu* Heppner (Fig. 1)	Heppner (1998)	**<0.001**	**<0.001**
3	Yponomeutidae s. l. (Fig. 1)	Moriuti (1977)	**<0.001**	**<0.001**
4	Yponomeutidae *sensu* Kyrki (Table 1)	Kyrki (1990)	**<0.001**	**<0.001**
5	Cedestinae	Friese (1960)	**<0.001**	**0.002**
6	Yponomeutidae B1 group	Friese (1960)	**0.001**	**0.001**
7	Plutellidae+Praydidae	Heppner (1998)	**<0.001**	**<0.001**
8	Plutellidae+*Scythropia*	Heppner (1998)	**<0.001**	**0.002**
9	Plutellidae *sensu* Heppner (Table 1)	Heppner (1998)	**<0.001**	**<0.001**
10	#9+*Ochsenheimeria*	Heppner (1998)	**<0.001**	**<0.001**
11	Lyonetiinae+Cemiostominae	Kyrki (1990)	0.259	0.180
12	Lyonetiidae+Bedelliidae	Kuroko (1964)	**0.005**	**0.005**

All analyses are based on the 8–27 gene nt123 and degen1 data sets. P values <0.05 in bold.

### Host Plant Associations and Biogeography

To explore the evolutionary history of Yponomeutoidea with respect to larval host plant associations and biogeography, we compiled data from the literature on these features for all described yponomeutoid species. Given current uncertainty about the limits of the superfamily, we considered only genera whose placements within Yponomeutoidea are secure. Host records were retrieved primarily from the HOSTS website [64]. These data were checked for possible error and supplemented by records from other sources. All suspicious records, possibly representing misidentification of larvae, misidentification of hosts, or confusion with adult-habitat association, were excluded. Individual host records were combined into lists of plant families or higher clades used by each of the 16 major yponomeutoid lineages identified on our molecular phylogeny. Higher classification of host plants follows APG III [65] for angiosperms and Fu et al. [66] for gymnosperms. Host ranges of individual yponomeutoid species were categorized as either oligophagous (feeding on plants in a single order) or polyphagous (feeding on plants in more than one order). The predominant growth form of hosts for each yponomeutoid lineage was categorized as arboreal (trees and shrubs), herbaceous, or scandent (vines and lianas), and alternatively as woody versus herbaceous. We also scored site and mode of feeding. Finally, for each lineage we tabulated the proportions of species and genera for which at least one host plant record is available, using species and generic diversity estimates from van Nieukerken et al. [2] or the first author’s unpublished data.

Information on yponomeutoid distributions across major biogeographical regions was assembled from global reviews (e.g. [67], [68], [69]) and local checklists (e.g. [20], [70], [71], [72], [73], [74]). Distributions due to human-caused dispersal (accidental or deliberate introduction) were excluded when discernable from non-anthropogenic causes. Data for individual species were compiled into summaries of numbers of species occurring in each region for each major yponomeutoid lineage, as described previously for host plant records. For species occurring in more than one region, each region was counted independently, thus some species were counted more than once. Our compilations are based primarily on described species, but undescribed species were included in several cases where they represent significant expansion of the known distribution of the lineage.

Generalization of host and distribution records by higher taxonomic groups often neglects variation, incompleteness, and bias in such data, introducing errors. For this reason, we did not attempt any formal statistical approach, although we did compute (by hand) parsimony optimizations of predominant feeding mode and host plant growth on a simplified version, reduced to major lineages, of the molecular phylogeny. Our goal was simply to provide a first phylogeny-based summary of evolutionary trends in yponomeutoid host-use evolution and biogeography.

## Results

**Figure 2:**
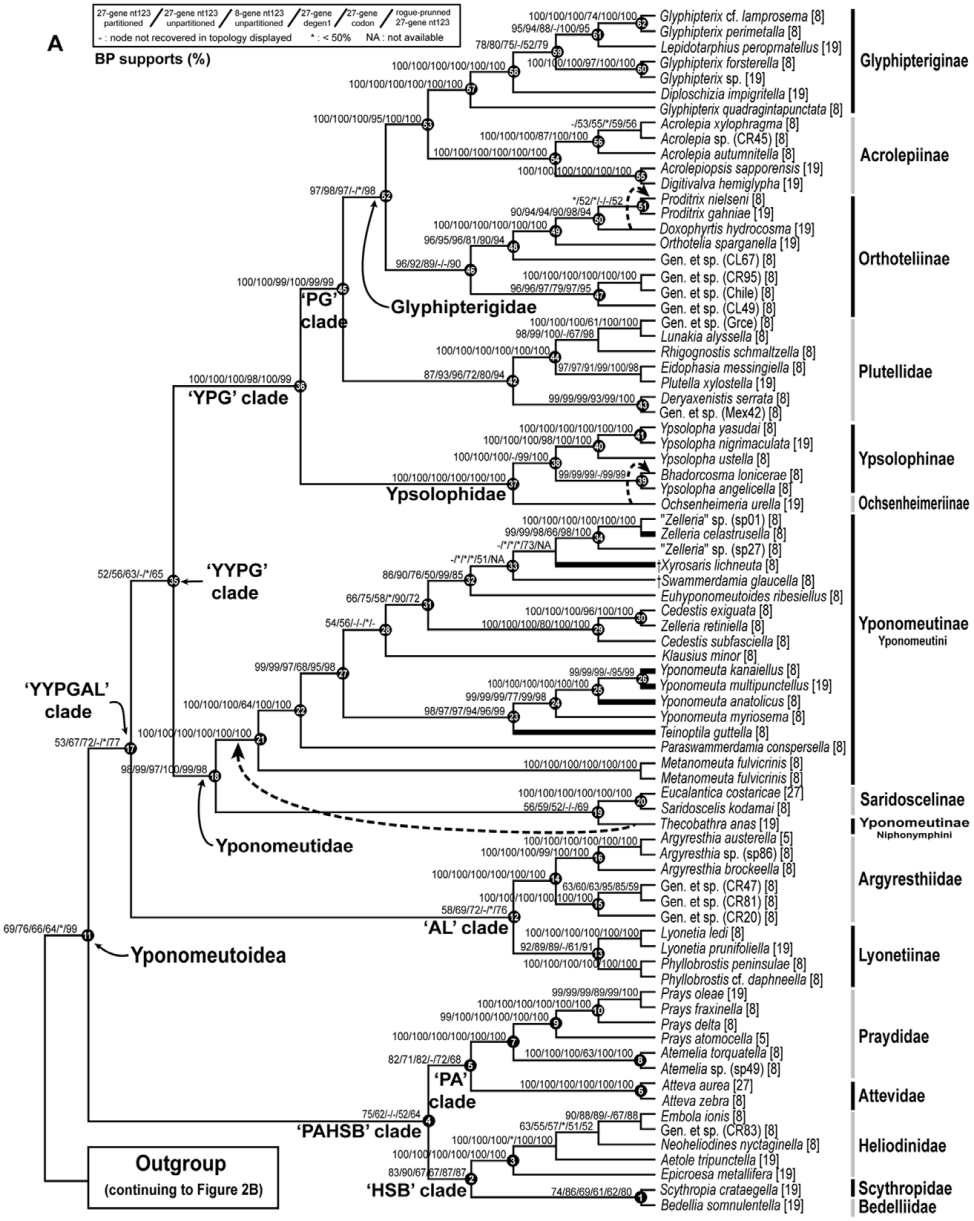
The best ML tree found for nt123 analysis of the deliberately incomplete 8–27 gene, 139-taxon data set, showing Yponomeutoidea only. Bootstrap supports shown above branches: partitioned 8–27 gene nt123/unpartitioned 8–27 gene nt123/8-gene nt123/8–27 gene degen1/8–27 gene codon model/rogue-pruned 8–27 gene nt123 (121 taxa). ‘−’ = node not recovered in the ML tree for that analysis. ‘*’ = bootstrap value <50%. ‘NA’ = bootstrap value undefined because data were obtained for ≤1 taxon in that clade for that analysis. Dotted lines indicate alternative topologies strongly supported by either degen1 or the codon model. Node numbers for selected nodes (solid circles) are provided to facilitate discussion. Thickened terminal branches denote yponomeutoid species feeding on Celastraceae.

**Figure 3:**
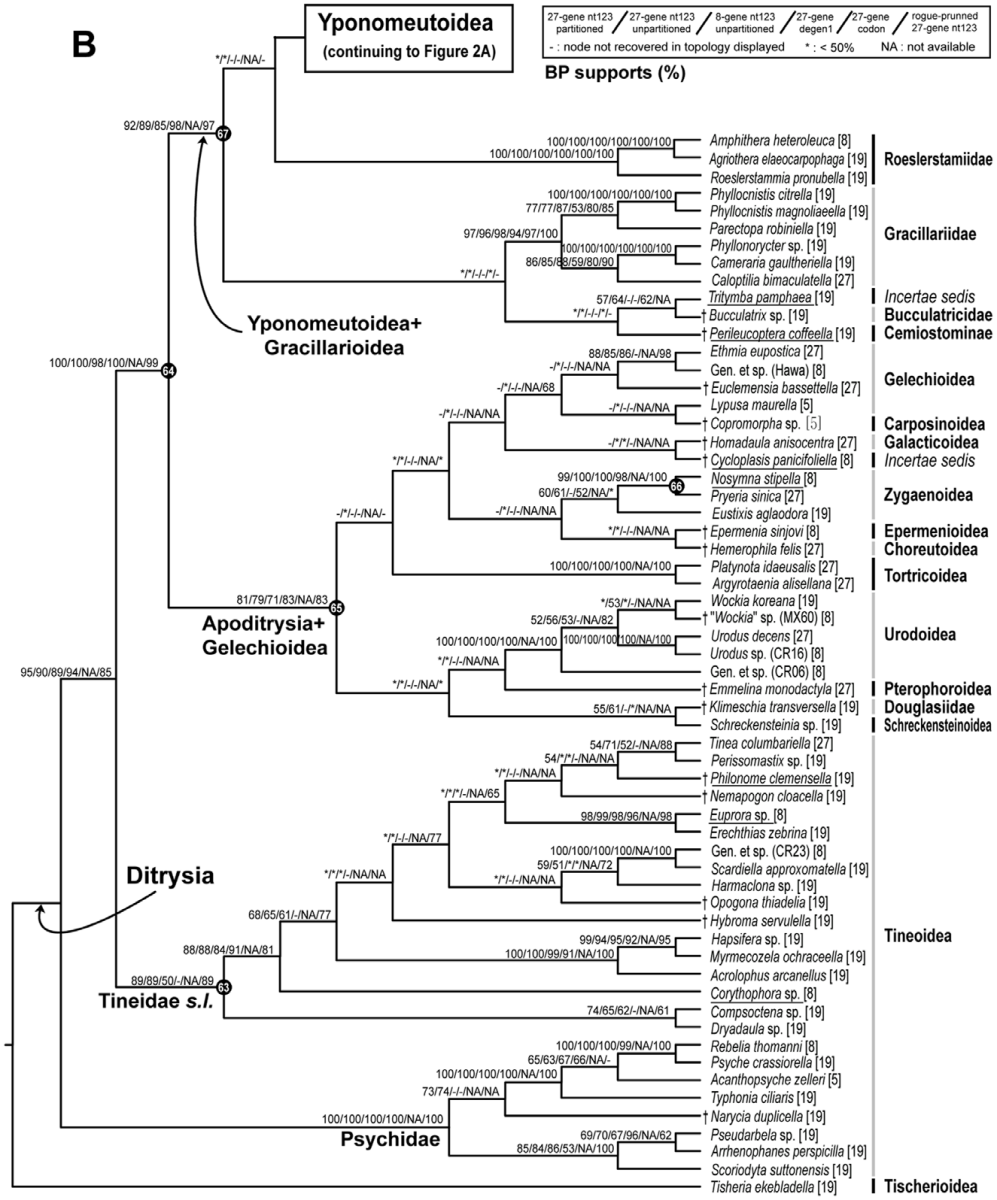
The best ML tree found for nt123 analysis of the deliberately incomplete 8–27 gene, 139-taxon data set (continued from Fig. 2), showing outgroups only. See Figure 2 for notes on bootstrap supports and node numbers. Terminal taxa shown in pink were initially thought to be yponomeutoids.

**Figure 4:**
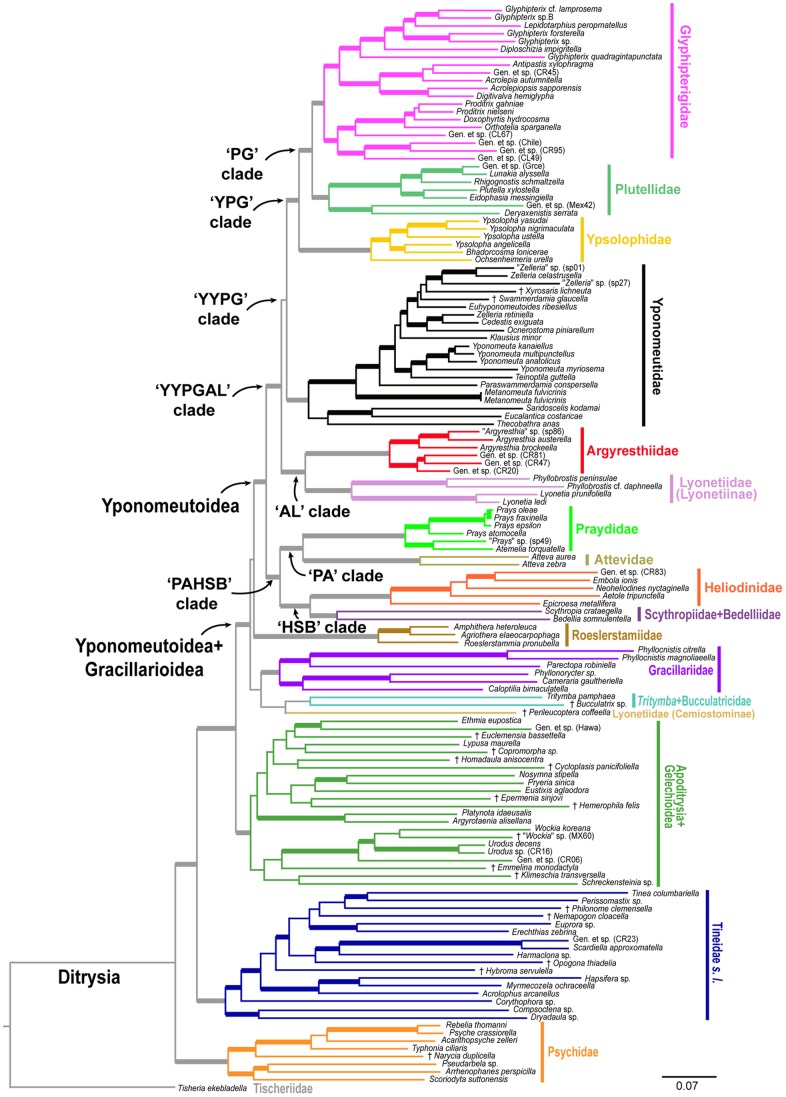
Phylogram representation of ML tree shown in Figures 2 and 3. Branch lengths are proportional to total number of substitutions per site. Thickened branches are supported by ≥70% bootstrap in at least one analysis summarized in Figures 2 and 3.

The best-score ML tree found in 150 GARLI searches for the 8–27 gene, 139-taxon nt123 analysis is shown in Figures 2 and 3. Figure 2 shows just the Yponomeutoidea as recovered here (79 taxa), while Figure 3 shows the outgroup region of the tree. Bootstrap values for five different combinations of character coding (nt123, nt123 partitioned, degen1) and gene sample (8 genes only vs. 8+19 genes), plus nt123 with rogue taxa removed, are superimposed on each node of this tree. Overall, the tree is well supported: 65 of the 78 nodes in Figures 2 and 3, or 83%, had strong bootstrap support (≥80%) from at least one analysis. Figure 4 shows the same topology in a phylogram format, with thickened branches denoting bootstrap support of ≥70% from at least one of the bootstrap analyses summarized in Figures 2 and 3.

The most robust phylogenies came from the nt123 analysis of the 8–27 gene deliberately incomplete data set (Fig. 2; Table 4). Within Yponomeutoidea (Fig. 2; 79 taxa) this analysis yielded 59 very strongly supported (BP≥90%), 4 strongly supported (BP = 80–89%) and 3 moderately supported (BP = 70–79%) nodes, for a sum of 66 nodes (of 78 total), or 85%, with BP≥70%. The results for the partitioned nt123 analysis were nearly identical: 58 nodes with BP≥90%, 4 nodes with BP = 80–89% and 3 with BP = 70–79%. The 8–27 gene degen1 analysis yielded 37 nodes with BP≥90%, 6 with BP = 80–89% and 4 with BP = 70–79%, for a total of 47/78 = 60% of nodes with BP≥70%. The codon model results were intermediate between those from nt123 and degen1 but closer to the former, with 54 nodes of BP≥90%, 3 of BP = 80–89% and 2 of BP = 70–79%, for a total of 59/78 = 76% of nodes with BP≥70%. The nt123 unpartitioned and nt123 partitioned trees were nearly identical, disagreeing at only three nodes weakly supported in each. The degen1 tree disagreed with the nt123 tree at 18 nodes, of which 8 were very strongly supported, 2 strongly supported, one moderately supported and 7 poorly supported (BP≤60%) in the nt123 tree. In only two cases, however, was a node strongly supported in the degen1 analysis but not present in the nt123 tree, while in no case was a node strongly supported in one tree and strongly contradicted in the other.

**Table 4:** Bootstrap supports for selected clades.

Node #	Selected Clade	4-genent123	8-genent123	8–27 genent123	8–27 genepartition	8–27 genedegen1	8–27 geneCodon	8–27 gene &no-rogue nt123
1	*Bedellia*+*Scythropia*	<50	69	86	74	61	62	80
2	‘H·S·B’ clade	56	67	90	83	67	87	87
3	Heliodinidae	100	100	100	100	100	100	100
4	‘P·A·H·S·B’ clade	–	–	62	75	–	52	64
5	‘P·A’ clade	96	82	71	82	–	72	68
6	Attevidae	100	100	100	100	100	100	100
7	Praydidae	100	100	100	100	100	100	100
8	*Atemelia*	100	100	100	100	100	100	100
9	*Prays*	89	100	100	99	100	100	100
11	Yponomeutoidea(excl. Cemiostomiinae)	–	66	76	69	64	<50	99
12	‘A·L’ clade	–	72	69	58	–	<50	76
13	Lyonetiidae (Lyonetiinae)	89	89	89	92	–	61	91
14	Argyresthiidae	100	100	100	100	100	100	100
15	“*Dasycarea*” group	100	100	100	100	100	100	100
16	*Argyresthia*	100	100	100	100	100	100	100
17	‘Y·Y·P·G·A·L’ clade	–	72	67	53	–	<50	77
18	Yponomeutidae	98	97	99	98	100	99	98
19	Saridoscelinae+*Theco-bathra*	<50	52	59	56	–	–	69
20	Saridoscelinae	100	100	100	100	100	100	100
21a	Yponomeutini	100	100	100	100	100	100	100
21b	Yponomeutini+ *Theco-bathra*	–	–	–	–	82	52	–
23	*Yponomeuta* group	99	97	97	98	94	96	99
29	*Cedestis*+*Zelleria* (part)	100	100	100	100	80	100	100
31	Node 29+32	–	58	75	66	<50	90	72
32	*Zelleria* (part)+*Xyrosaris*+ *Swammerdamia*+*Euhypo-nomeutoides*	–	76	90	86	50	99	85
35	‘Y·Y·P·G’ clade	–	63	56	52	–	<50	65
36	‘Y·P·G’ clade	96	100	100	100	98	100	99
37	Ypsolophidae	100	100	100	100	100	100	100
38	Ypsolophinae	100	100	100	100	–	99	100
39	*Bhadorcosma*+*Ypsolopa angelicella*	96	99	99	99	–	99	99
42	Plutellidae	92	96	93	87	72	80	94
43	*Deryaxenistis* group	97	99	99	99	93	99	100
44	Core Plutellidae	100	100	100	100	100	100	100
45	‘P·G’ clade	95	99	100	100	100	99	99
46	Orthoteliinae	86	89	92	96	–	–	90
47	Neotropical Orthoteliinae	99	96	96	97	79	97	95
48	Core Orthoteliinae	90	96	95	96	81	90	94
51a	*Proditrix*	<50	<50	52	<50	–	–	52
51b	*Doxophytis*+*Proditrix nielseni*	–	–	–	–	86	56	–
52	Glyphipterigidae	98	97	98	97	–	<50	98
53	Glyphipteriginae+Acro-lepiinae	96	100	100	100	95	100	100
54	Acrolepiinae	100	100	100	100	100	100	100
57	Glyphipteriginae	100	100	100	100	100	100	100
59	*Glyphipterix* (part)+*Lepi-dotarphius*	–	75	80	78	–	52	79

Dashes indicate unrecovered clades. Node numbers corresponding to Figure 2 (a & b for alternative topologies).

The 8-gene and 8–27 gene nt123 trees were almost entirely congruent, differing in only 2 weakly supported nodes. Of the matching nodes between the two analyses, 12 were better supported in the 8-gene analysis, with a mean difference of +3.33% and a range of 1–11%, while the 19+ gene analysis yielded higher support at 16 nodes, with a mean difference of +7.56% and a range of 1–23%. The 8-gene analysis yielded 55 nodes with BP≥90%, 5 with BP = 80–89% and 3 with BP = 70–79%, for a total of 63/78 = 81% of nodes with BP≥70%, only slightly lower than the 19+ gene analysis. However, a few nodes showed substantial increase in support with increased gene sampling. Among these are three that subtend multiple families: Heliodinidae+Bedelliidae+*Scythropia* (Fig. 2, **node 2**; BP = 90/67, 19+ genes/8 genes); Bedelliidae+*Scythropia* (Fig. 2, **node 1**; BP = 86/69); and Yponomeutoidea (Fig. 2, **node 10**; BP = 76/66).

Our rogue taxon analysis using RogueNaRok [58] identified 16 rogue taxa for the 8–27 gene nt123 data set as a whole (Table 2). All but one (Yponomeutidae: *Xylosaris lichineuta*) proved to lie among the outgroups, although several others were thought by some previous authors to belong to Yponomeutoidea (Table 2). Two additional rogue taxa, both yponomeutoids (Lyonetiidae: *Perileucoptera* and Yponomeutidae: *Swammerdamia*), were discovered when only Yponomeutoidea and Gracillarioidea were analyzed. We found no significant correlation between rogue status and sequence data incompleteness (Table 2: SC index). Removal of the 18 rogue taxa resulted in increased bootstrap values for 14 nodes and decreases for 17 nodes in the tree for Yponomeutoidea (Fig. 2). However, 77% of these changes were very small (≤3%). When only changes of >3% are counted, there are just two decreases in support in the rogue-pruned analysis, one of 5% and one of 6%. In contrast, five nodes showed increases, ranging from 7% to 23%. Among the nodes undergoing the strongest improvements in support are Yponomeutoidea (Fig. 2, **node 10**; BP = 99/76, after/before rogue removal); the YPGAL clade (Fig. 2, **node 16**; BP = 77/67); and the AL clade (Fig. 2, **node 11**; BP = 76/69). Half of the increase in bootstrap values across all affected nodes can be explained by deletion of *Perileucoptera coffeella* alone (data not shown).

## Discussion

### Phylogenetic Signal Sources, Partial Gene Sample Augmentation and Rogue Taxon Analysis

Our results exemplify the ability of combined analyses of multiple genes to produce robust phylogeny estimates even when there is little strong signal from any individual gene [75]; none of the deeper nodes with substantial support (BP≥70) in the concatenated analysis (Fig. 2) were strongly supported by any of the initial 8 genes (Figure S5) or the 11 additional genes sampled for a subset of taxa (data not shown). The utility of concatenated analysis can be undermined when individual gene trees conflict with each other or with the species tree [76]. Our individual gene trees showed little evidence of strong conflict (Figure S5), reinforcing the value of combined analysis for this data set, and implying that the low to modest support for some “backbone” nodes is not in general the result of conflict among gene trees. In a few instances noted below, however, there is indirect evidence that inter-gene conflict may be influencing bootstrap values.

We also see minimal evidence overall of spurious signal resulting from heterogeneity and convergence in base composition. Compositional heterogeneity is especially common at sites undergoing synonymous substitution [75], and our data are no exception; there is highly significant variation in composition across taxa in both nt3 and nt1+nt2, while heterogeneity is minor with synonymous differences removed (the degen1 data set). Conflicting signal due to compositional heterogeneity, in addition to substitutional saturation, may contribute to the inability of nt3 alone (Figure S4) to provide notable support to *any* of the among-family relationships that receive moderate to strong bootstraps from the full data set (nt123), despite providing a great majority of the total evolutionary change inferred from that data set and strongly supporting many individual families and sub-clades thereof. If composition had major effects on phylogenetic inference, however, we might expect to see repeated instances of conflicting moderate to strong bootstrap values between the total data set (nt123), dominated by synonymous change, and non-synonymous change only, as estimated by the degen1 analysis. No such cases were found, although several examples of lesser conflict are pointed out below. Rather than conflicting, the signals from synonymous and non-synonymous change appear to be largely complementary.

Our results provide another instance in which deliberately unequal gene sample augmentation markedly improves support for deeper nodes without introducing any apparent artifacts due to large blocks of non-random missing data. Nt123 analyses of the 8-gene “complete” matrix (27% inadvertently missing data due to sporadic failures of amplification or sequencing) and the deliberately-incomplete 8–27 gene matrix (55% missing data) yielded nearly identical topologies and similar bootstrap values. The 8–27 gene analysis produced higher support overall, however, and markedly increased bootstraps for several deeper nodes, including Yponomeutoidea (Fig. 2, **node 10**). Similar findings have been reported in several recent studies of Lepidoptera [30], [33], [35].

The potential for even a few “rogue” taxa to substantially reduce bootstrap support, obscuring otherwise strong signal on relationships among the remaining taxa, is now widely recognized [77], [78]. Despite multiple proposals, however, it has been unclear how to best identify such taxa and evaluate their effect. We believe that the RogueNaRok procedure of Aberer et al. [58] is an important advance toward solving this problem. It sets out a very reasonable and explicit optimality criterion for deciding which and how many potential rogue taxa should be removed, balancing the increased support gained by deleting those taxa against the information lost through their deletion, and provides well-tested heuristic algorithms for estimating an optimal set of taxa to delete. Application of RogueNaRok following our 8–27 gene, 139-taxon nt123 analysis identified 18 rogue taxa meriting deletion. Removal of these taxa resulted in substantial bootstrap support increases for five nodes, most notably an increase from 76 to 99% for Yponomeutoidea. We predict that RogueNaRok will prove widely useful in phylogenetic studies of large taxon sets.

### Monophyly, Composition and Phylogenetic Position of Yponomeutoidea

**Figure 5:**
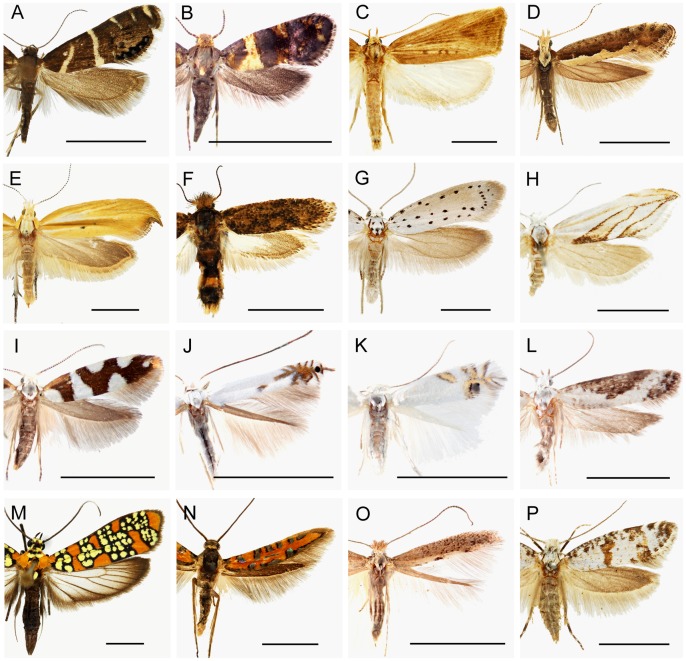
Representative adult habitus images of all yponomeutoid families and subfamilies recognized in this study. Scale bar = 5 mm. A. Glyphipterigidae: Glyphipteriginae, *Glyphipterix bifasciata* (Walsingham); B. Glyphipterigidae: Acrolepiinae, *Acrolepia xylophragma* (Meyrick); C. Glyphipterigidae: Orthoteliinae, *Orthotelia sparganella* (Thunberg); D. Plutellidae, *Plutella xylostella* (Linnaeus); E. Ypsolophidae: Ypsolophinae, *Ypsolopha blandella* (Christoph); F. Ypsolophidae: Ochsenheimeriinae, *Ochsenheimeria vacculella* Fisher von Roeslerstamm; G. Yponomeutidae: Yponomeutinae, *Yponomeuta padellus* Linnaeus; H. Yponomeutidae: Saridoscelinae, *Saridoscelis kodamai* Moriuti; I. Argyresthiidae, *Argyresthia brockeella* (Hübner); J. Lyonetiidae: Lyonetiinae, *Lyonetia ledi* Wocke; K. Lyonetiidae: Cemiostominae, *Leucoptera spartifoliella* (Hübner); L. Praydidae, *Prays fraxinella* (Bjerkander); M. Attevidae, *Atteva aurea* (Fitch); N. Heliodinidae, *Embola ciccella* (Barnes et Busck); O. Bedelliidae, *Bedellia somnulentella* (Zeller); P. Scythropiidae **stat. rev.**, *Scythropia crataegella* (Linnaeus).

**Figure 6:**
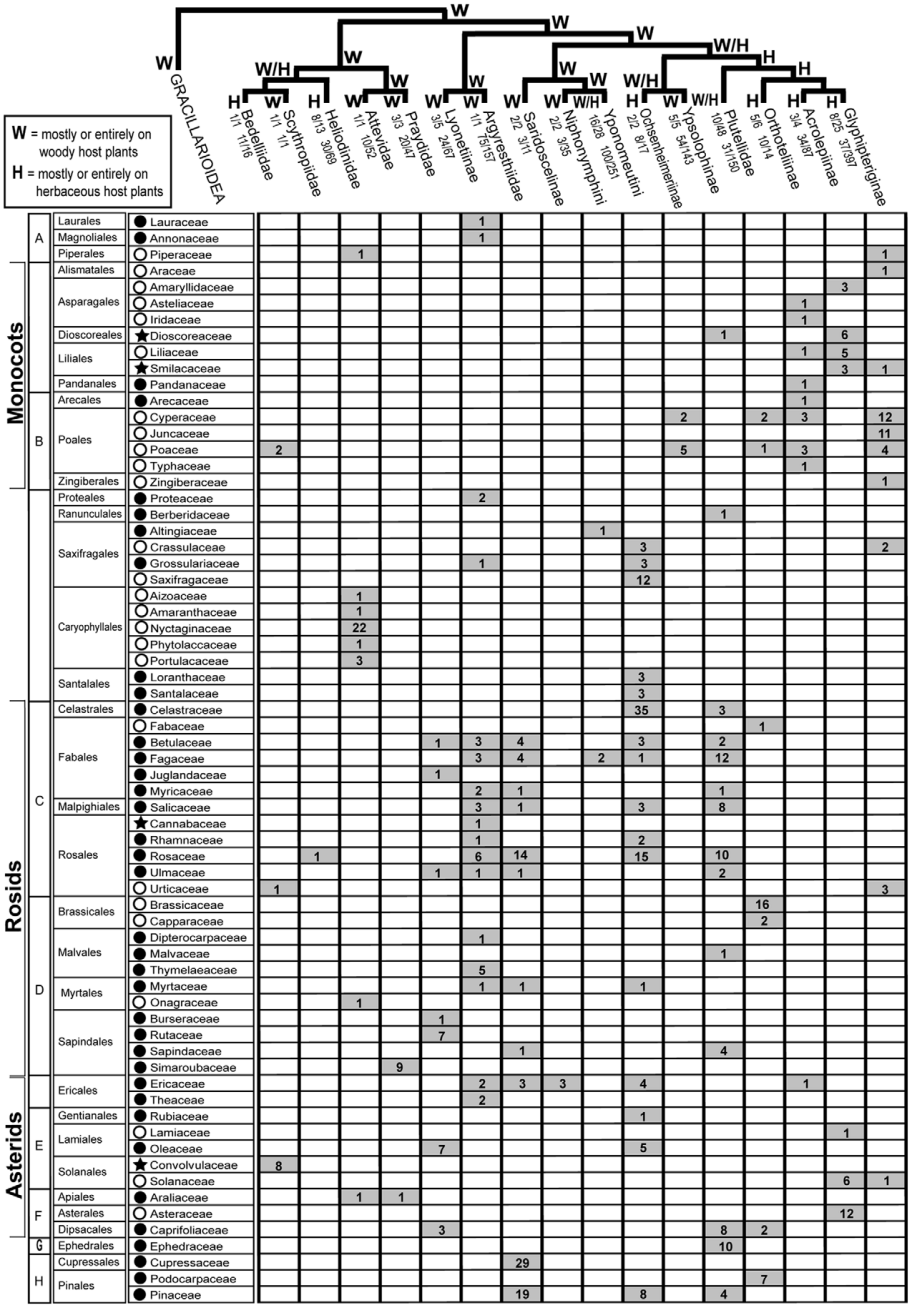
Host plant families of 16 major yponomeutoid lineages. The cladogram is simplified from figure 2, annotated with predominant growth form of host plants (‘W’ for woody plants vs. ‘H’ for herbaceous plants). Fractions below yponomeutoid taxon names denote host record completeness for genera and species (in that order), calculated from the number of genera or species with host records relative to the total number of known genera or species. Host plant families used by each lineage are denoted by gray cells showing the numbers of species feeding on that plant family. Symbols denote the dominant growth-forms of each plant family: shaded circles = trees and shrubs; open circles = herbs; and shaded stars = veins and lianas. Capital letters next to host plant orders denote membership in clades above the order level: A – magnoliids, B – commelinids, C – fabids, D – malvids, E – lamiids, F – campanulids, G – Gnetophyta, and H – Pinophyta.

**Figure 7:**
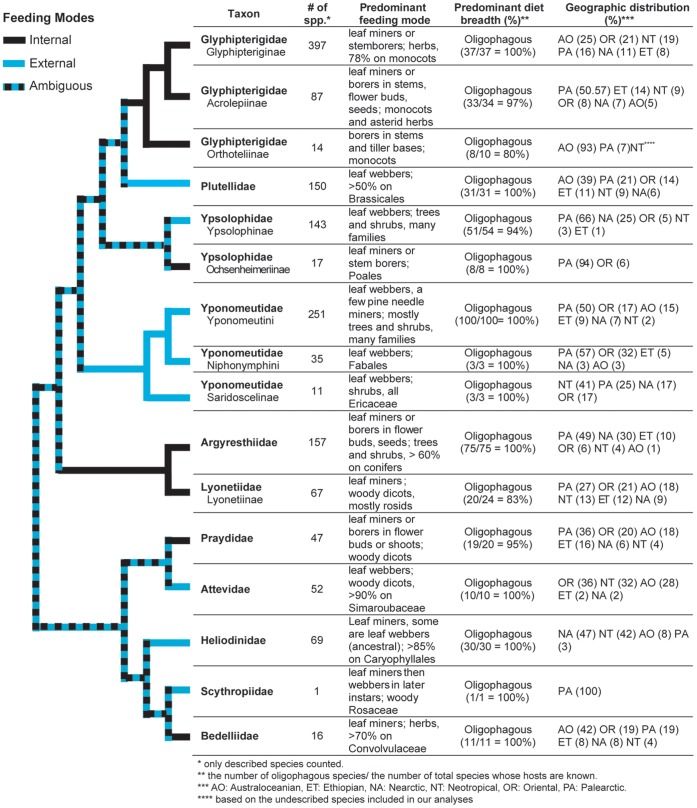
Species diversity, feeding mode, diet breadth and geographic distribution of 16 major yponomeutoid lineages. The tree topology is that of Figure 6. Branch colors indicate predominant feeding modes: black = internal feeding; blue = external feeding; alternating black and blue = state ambiguous under parsimony optimization.

In this and subsequent sections we evaluate the implications of our molecular results for current understanding of the phylogeny of yponomeutoids, and for their classification. Our exposition proceeds from the base to the tips of the tree in Figure 2, and makes repeated reference to the node numbers labeled on that tree. Representative adult habitus images for nearly all of the 16 families and subfamilies discussed below are provided in Figure 5. The species diversities, geographic distributions and larval feeding habits of these families and subfamilies are summarized in Figures 6 and 7.

All of our molecular analyses support monophyly for Yponomeutoidea (Fig. 2, **node 11**) in approximately the sense of Kyrki [25], [26]. Bootstrap support is moderate (BP = 76%, nt123) for the full data set but rises to very strong (BP = 99, nt123) when the 18 rogue taxa are removed. Kyrki [25] initially proposed a single synapomorphy for Yponomeutoidea, the presence of posterior expansions on the 8^th^ abdominal pleuron (“pleural lobes”) in males. He later added another possible synapomorphy, a transverse ridge on the second abdominal sternite [26]. On this basis he included seven families: Yponomeutidae, Plutellidae (including Acrolepiidae, later separated by Dugdale et al. [31]), Ypsolophidae, Glyphipterigidae, Heliodinidae, Lyonetiidae, and Bedelliidae. This hypothesis had been questioned because it requires independent losses of the two synapomorphies in some of the included groups [31]. In our results, the main remaining question about the composition of Yponomeutoidea concerns Lyonetiidae. Our analyses always separate Lyonetiinae from Cemiostominae, placing the former inside Yponomeutoidea but the latter outside, among the gracillarioids. However, the position of *Perileucoptera*, our sole cemiostomine, is exceptionally unstable. It is identified as a rogue taxon by the RNR analysis, and our AU test cannot reject the monophyly of Lyonetiidae (Table 3).

Among the out-groups included in our analyses, Gracillarioidea sensu van Nieukerken et al. [2], i.e. with Douglasiidae excluded, were strongly supported (Fig. 3, **node 67**; BP 85–97, all analyses) as the closest relatives to Yponomeutoidea. This clade has been strongly supported in almost all previous molecular studies (e.g. [28], [30], [35]). However, the deeper divergences within Yponomeutoidea+Gracillarioidea (the G.B.R.Y. clade of Kawahara et al. [35]) are very weakly supported. Like Kawahara et al. [35], we find no molecular evidence for monophyly of Gracillarioidea. Eventually it may be reasonable to merge Gracillarioidea into an Yponomeutoidea *sensu lato,* but such a change is beyond the scope of the present study.

Our results support several earlier morphology-based proposals that excluded a variety of taxa from membership in, or close relatedness to, Yponomeutoidea. Galacticoidea, Urodoidea and Schreckensteinioidea, once placed in Yponomeutoidea [79], [80], [81], [82], are decisively excluded from Yponomeutoidea+Gracillarioidea, here (Fig. 3, **node 67**) and in all other recent molecular studies. Removal of the putative yponomeutid genus *Nosymna* Walker, 1864 to Zygaenoidea by Heppner [83] is also confirmed by our analyses (Fig. 3, **node 66**), as is the exclusion of *Cycloplasis* Clemens, 1864 from Heliodinidae by Hsu and Powell [84]. Our results place *Cycloplasis* in Apoditrysia+Gelechioidea (Fig. 3, **node 65**; BP = 71–83, all analyses). Two genera previously placed in Lyonetiidae, *Philonome* Chambers, 1872 and *Corythophora* auct Braun, 1915, are here strongly supported as belonging to Tineoidea (Fig. 3, **node 63**; BP = 90, nt123).

### Basal Split within Yponomeutoidea

Within Yponomeutoidea (Fig. 2, **node 11**), our results provide moderate to strong support for most nodes above the family level, allowing us to construct a working hypothesis of higher phylogeny across the superfamily. In presenting this hypothesis below, we make repeated use of informal clade names based primarily on the first letters of the names of the included families.

In the tree of Fig. 2, the basal split is between a ‘PAHSB clade’ (Fig. 2, **node 4**; maximum BP = 75, nt123 partitioned) consisting of Praydidae, Attevidae, Heliodinidae, Bedelliidae and *Scythropia*, and a ‘YYPGAL clade’ (Fig. 2, **node 17**; maximum BP = 77, rogue-pruned nt123) consisting of Yponomeutidae, Ypsolophidae, Plutellidae, Glyphipterigidae, Argyresthiidae and Lyonetiidae. Because bootstrap support for these clades is modest at best, and they are contradicted, albeit very weakly, by degen1, we regard them as provisional. Neither clade has ever been proposed on the basis of morphology. However, our working hypothesis, including this basal split, fits the molecular data much better than any of the alternative proposals for among-family relationships shown in Figure 1, all of which are decisively rejected (P<0.001) by the AU test (Table 3).

### Relationships within the PAHSB Clade

This clade (Fig. 2, **node 4**), for which no morphological synapomorphies are yet known, contains five relatively small yponomeutoid groups. It divides basally into a ‘PA clade’ (Fig. 2, **node 5**; maximum BP = 82, nt123 partitioned) containing the Praydidae and Attevidae, and an ‘HSB clade’ (Fig. 2, **node 2**; BP = 90, nt123) consisting of Heliodinidae, Bedelliidae and *Scythropia*. The latter was previously treated as a subfamily of Yponomeutidae.

The PA clade receives moderate to strong support from nearly all of our analyses, except that it is very weakly contradicted by degen1 (BP≤38). The groups based on *Prays* and *Atteva*, here treated as families following van Nieukerken et al. [2], were treated as subfamilies of Yponomeutidae by Kyrki [26], while others have regarded the *Prays* group as closer to Plutellidae than to Yponomeutidae [20], [27], [85]; Heppner [1] treated it as a subfamily of Plutellidae. All of these hypotheses are strongly contradicted by our results.

While previous ideas about their phylogenetic position receive no support, the molecular data do corroborate Kyrki’s [26] assertion of a close relationship between the *Prays* and *Atteva* groups, based on two synapomorphies, the lack of a pecten on the antennal scape and the presence of a larval cranial seta P_1_ that lies on or above the line defined by setae Af_2_–P_2_. A possible additional synapomorphy is the presence of less than four segments in the maxillary palp. Ulenberg [86] also recovered the pairing of the *Prays* and *Atteva* groups within Yponomeutidae, in a parsimony analysis using Kyrki’s [26] characters. These putative synapomorphies might be doubted because they are reductions or homoplasious, but the molecular results suggest that they are real. We nonetheless treat these groups as separate families because the molecular evidence is not yet completely incontrovertible.

Monophyly of the Praydidae, here represented by *Prays* and *Atemelia*, is very strongly supported by our data (Fig. 2, **node 7**; BP = 100, all analyses). The members of this group are easily distinguished from other yponomeutoids by an unusually broad male 8^th^ sternum and by female apophyses anteriores lacking a branched costa at the base [20], [27]. Our data also strongly resolve the relationships among the four *Prays* species sampled (Fig. 2, **nodes 9, 10**; BP = 89–100, all analyses). Praydidae, comprising 3 genera and 47 species, are a cosmopolitan group that is most diverse in the Old World. The larvae are initially endophagous feeders in leaves, buds or shoots of woody dicots of diverse families; in some species, older larvae feed externally in webs [31].

The two species of *Atteva* included in our sample are likewise strongly grouped (Fig. 2, **node 6**; BP = 100). The Attevidae can be defined by four autapomorphies [25]: the presence of chaetosema; reduction of the hindleg tibia and tarsus, especially in the male; the presence of two subventral setae on the larval meso- and metathorax; and concealment of the labial palps in the pupa. Attevidae are a predominantly pan-tropical group of 52 described species in a single genus *Atteva*, most diverse in the Oriental region. The larvae are communal leaf webbers on woody dicots, with >90% of records from Simaroubaceae [31].

Monophyly of the probable sister group to the PA clade, the HSB clade (Fig. 2, **node 2**; maximum BP = 90, nt123), is supported by all of our analyses. The grouping of Heliodinidae, Bedelliidae and *Scythropia* has not been previously proposed. The closest antecedents are the grouping of Heliodinidae, Bedelliidae and Lyonetiidae by Kyrki [26] and that of Lyonetiidae (including Bedelliinae), Acrolepiidae, and Heliodinidae by Heppner [1]. Kyrki [26] proposed three possible synapomorphies for Heliodinidae+Bedelliidae: larva with a long spinneret; larval seta V_1_ not apparent on the thorax; and pupa without a cocoon. It is not known whether *Scythropia* shares any of these traits. The search for morphological synapomorphies of the strongly-supported HSB clade merits further effort.

The molecular data strongly favor monophyly for Heliodinidae as sampled here (Fig. 2, **node 3**; BP = 100, all analyses), corroborating the re-definition of this family by Hsu and Powell [84]. Kyrki [25] suggested four synapomorphies for heliodinids: in the adult, smooth scaling on the head and absence of the CuP vein in forewing; and in the pupa, strong lateral ridges and stiff, long lateral and dorsal bristles. Only the last trait, however, is limited to the re-defined Heliodinidae. In their cladistic analyses, Hsu and Powell [84] found three additional synapomorphies: female apophyses anteriores with ventral branches originating from a fused medial sclerite; male tegumen greatly expanded posteriorly, forming a conical or tubular sclerotized sac; and the forewing M vein with two branches. Adult diurnality is another possible synapomorphy [31]. Our data strongly resolve two of the three nodes subtending the five heliodinid genera sampled and yield relationships among these genera that are entirely concordant with the morphological cladistic analysis of Hsu and Powell [84]. Heliodinidae are a widespread but primarily New World group of 13 genera and 69 described species [2]. The larvae are variable in feeding habits, with most species feeding internally in leaves, stems or fruits, while others are externally-feeding leaf webbers, all on herbaceous plants. The great majority of records (>85%) are from Caryophyllales, primarily Nyctaginaceae [84].

The apparent sister group to Heliodinidae is the strongly supported pairing of *Bedellia*+*Scythropia* (Fig. 2, **node 1**), favored in all of our analyses, with bootstraps as high as 86% (8–27 gene nt123). This is an entirely new hypothesis. No morphological synapomorphies are apparent, but a search for these would be worthwhile, given the strength of the molecular evidence. Bedelliidae are often confused with Lyonetiidae or Gracillariidae (see [87] for detailed history). Heppner [88] recently transferred *Philonome* and *Euprora* to Bedelliidae (Bedelliinae auct), but our analyses very strongly place these genera in Tineidae instead (Fig. 3). Kyrki [25], [26] maintained separate family status for *Bedellia.* The widespread contrasting view, that *Bedellia* constitutes a subfamily of Lyonetiidae [1], [87], [89], [90], is unsupported by clear morphological synapomorphies and is likewise strongly rejected by our analyses, including the AU test (Table 3, #12). Bedelliidae are a monogeneric, cosmopolitan group of 16 species, most diverse in the Old World [2]. The larvae are leaf miners in herbaceous plants, with 70% of records from Convolvulaceae [31].

The position of *Scythropia* has likewise been controversial. Kyrki [26] suggested that it constitutes the first-diverging subfamily of Yponomeutidae, while others, such as Friese [20], Moriuti [27], and Heppner [1], grouped this genus with Plutellidae. Our results strongly contradict all previous hypotheses about the systematic position of *Scythropia*. We are reluctant to combine it with Bedelliidae, given the current complete absence of morphological support for such a pairing, and therefore hereby elevate Scythropiinae to Scythropiidae **stat. rev.** Larvae of the single, Palearctic species, *Scythropia crataegella*, are initially leaf miners and subsequently feed externally in a communal web, on *Crataegus* and sometimes other woody Rosaceae [31].

### Relationships within the YYPGAL Clade

The majority of yponomeutoid species belong to the provisional YYPGAL clade (Fig. 2, **node 17**). This group is monophyletic in all analyses except degen1, where it is only very weakly contradicted (BP<20; tree not shown). However, bootstrap support is moderate at best (BP = 77, rogue-pruned nt123). Limited support for this node may result in part from conflict among gene trees, as suggested by the fact that the bootstrap value for 8–27 genes is lower than that for 8 genes (67 vs. 72%). No grouping like the YYPGAL clade has been proposed previously, and no morphological synapomorphies are apparent.

Within the YYPGAL clade there are three main sub-clades, each with moderate or strong support: an ‘AL clade’ consisting of Argyresthiidae and Lyonetiidae (Fig. 2, **node 12**; maximum BP = 76, rogue-pruned nt123); Yponomeutidae (Fig. 2, **node 18**; BP≥97, all analyses); and a ‘YPG clade’ consisting of Ypsolophidae, Plutellidae and Glyphipterigidae (Fig. 2, **node 36**; BP≥97, all analyses). Relationships among these three entities, however, are less clear. All analyses favor grouping of Yponomeutidae plus the YPG clade to the exclusion of the AL clade (Fig. 2, **node 35**), with the weakly supported exception of degen1. However, bootstrap support for this relationship never exceeds 65%, and is higher for 8 genes than for 8–27 (63 versus 56%), again suggesting the presence of inter-gene conflict.

### Relationships within the AL Clade

The AL clade (Fig. 2, **node 12**) comprises Argyresthiidae plus Lyonetiidae: Lyonetiinae. It is monophyletic in all of our analyses except degen1, where it is only very weakly contradicted (BP<20; tree not shown). However, bootstrap support is moderate at best (BP = 77, rogue-pruned nt123). Limited support for this node may result in part from conflict among gene trees, as suggested by the fact that the bootstrap value for 8–27 genes is lower than that for 8 genes (69 vs. 72%). Grouping of these two taxa has never been proposed previously, and no morphological synapomorphies are apparent. In view of all the evidence, we regard this clade as only provisionally established. However, Kyrki’s [26] inclusion of Argyresthiidae as a subfamily of Yponomeutidae can be confidently ruled out.

Monophyly for Argyresthiidae as sampled here is very strongly supported (Fig. 2, **node 13**; BP = 100, all analyses). The family had been thought to be monobasic, defined by unique features of the male genitalia including a laterally produced vinculum and sensilla ornaments on the socii [31]. Our results, however, very strongly favor inclusion of a well-supported clade of several Neotropical yponomeutoids (Fig. 2, **node 15**; BP = 100, all analyses) that were originally assigned to, but later excluded from, Acrolepiinae [69]. These species are morphologically divergent from typical *Argyresthia*, which will necessitate a reevaluation of the currently hypothesized argyresthiid synapomorphies. Argyresthiidae are a cosmopolitan group of 157 described species, most species-rich in the Holarctic. The larvae are typically leaf miners or borers in flower buds, seeds or twigs of trees and shrubs [31]. About half of the records are from conifers.

Monophyly of the subfamily Lyonetiinae as sampled here (Fig. 2, **node 13**), comprising two species each of *Lyonetia* and *Phyllobrostis*, is supported by all but one of our analyses, with bootstraps up to 92%, although the two genera are separated by several nodes in the degen1 tree (BP≤64). A close relationship between *Lyonetia* and *Phyllobrostis*, to the exclusion of *Leucoptera* (Cemiostominae), was also supported by a cladistic analysis of morphology [91]. Lyonetiinae are a cosmopolitan group of 5 genera and 67 described species [2]. The larvae are typically leaf miners on woody dicots, of diverse families [31].

The Cemiostominae, in contrast, are one of the most problematic groups in our study. *Perileucoptera*, our sole representative, was identified as a rogue taxon. Cemiostomines differ from Lyonetiinae in many features, e.g. in having shorter antennae, different forewing pattern elements, and spine-like setae on the adult abdomen, leading some authors (e.g. [19], [92]) to place them in their own family. Kyrki [26], however, proposed uniting Cemiostominae and Lyonetiinae into a single family, citing as a possible synapomorphy the shared possession of an “eye cap” formed by scales on the antennal scape. Our molecular analyses nearly always separated the two subfamilies, excluding Cemiostominae but not Lyonetiinae from Yponomeutoidea, concordant with the view of Börner [19]. However, bootstrap support for Yponomeutoidea is modest at best except when *Perileucoptera* is excluded from the analysis, and support for alternative positions among the Gracillarioidea for *Perileucoptera* had very low support. Moreover, the four-gene nt123 analysis (Figure S2) grouped Lyonetiinae with Cemiostominae, albeit with very weak support. Finally, our AU test cannot reject the monophyly of Lyonetiinae+Cemiostominae as sampled here (Table 3: # 11). Mutanen et al. [29] also failed to recover Cemiostominae (represented by *Leucoptera*)+Lyonetiinae. Their analysis places *Leucoptera* as sister group to *Atteva* with 76% bootstrap support. Given the weak and conflicting molecular evidence on the placement of *Perileucoptera*, we tentatively retain Cemiostominae as a subfamily of Lyonetiidae pending further investigation. Although the composition of this family remains in doubt, our results do strongly confirm Kyrki’s [25] placement of Lyonetiidae in or near Yponomeutoidea: both subfamilies fall within the strongly supported clade Yponomeutoidea+Gracillarioidea (Fig. 3, **node 67**; BP 85–97, all analyses). The Cemiostominae are a cosmopolitan group of about 6 genera and 120 described species; the larvae are typically leaf miners in woody dicots of diverse families [31].

### Composition of and Relationships within Yponomeutidae

Different authors have hypothesized very different compositions for Yponomeutidae (Table 1). Our analyses very strongly support a circumscription of this family (Fig. 2, **node 18**; BP = 97–100, all analyses) that corresponds exactly to Yponomeutinae sensu Moriuti [27]. Moriuti [27] proposed two synapomorphies for this group, the presence of spine-like setae on the adult abdominal tergites, and a seta V_1_ on the larval head that is as large as a long tactile seta. Kyrki ([26], and see also [86]), in contrast, assigned six subfamilies to Yponomeutidae, three of which are now the separate families Argyresthiidae, Attevidae and Praydidae [2]. Kyrki’s hypothesis for Yponomeutidae has gained little support even from other morphological studies [31], and is soundly rejected by our AU test (Table 3: # 4). Yponomeutidae as delimited here are a cosmopolitan group of 32 genera and 297 described species, most diverse in the Palearctic. The larvae are usually communal leaf webbers, although some species of *Zelleria* mine pine needles [31]. A very diverse array of host families is used, mostly woody but some herbaceous.

Within his concept of Yponomeutinae, here treated as a family (Fig. 2, **node 18**), Moriuti [27] recognized two tribes, Yponomeutini and Saridoscelini, which we treat as subfamilies. One of these, here treated as Saridoscelinae, was previously restricted to *Saridoscelis*. The molecular data, however, very strongly indicate that *Saridoscelis* is the sister group to *Eucalantica*, an yponomeutoid genus of previously unsettled position (Fig. 2, **node 20**; BP = 100, all analyses). We therefore hereby re-define Saridoscelinae to include *Eucalantica*. Moriuti [27], followed by Kyrki [26] and Dugdale et al. [31], proposed two synapomorphies for *Saridoscelis*, a unique modification of the male 8^th^ abdominal sternite, and the presence of three branches in the M vein of the hindwing. In *Eucalantica* the condition of the male 8^th^ abdominal sternite is ambiguous; it may or may not share a derived modification with *Saridoscelis*. The number of hindwing M veins is sufficiently homoplasious in Yponomeutoidea that this character too is ambiguous evidence on the grouping of these two genera (J. Sohn, unpublished). Thus, further search is needed for morphological synapomorphies of the Saridoscelinae as here re-defined.

Within his concept of Yponomeutini, here treated as a subfamily, Moriuti [27] recognized two subtribes, here treated as the tribes Yponomeutini and Niphonymphini. The molecular evidence on monophyly of Yponomeutinae as defined here is somewhat complex due to conflicting results regarding the position of our representative of Niphonymphini, *Thecobathra*. In the nt123 and nt123 partitioned analyses, *Thecobathra* groups with Saridoscelinae, but with weak support (Fig. 2, **node 19**; BP 51–59). On the other hand, analyses emphasizing non-synonymous change (degen1 and codon model) place it as sister group to Yponomeutini, with strong support (BP = 82, degen1). Previous morphological studies have also supported monophyly for Niphonymphini+Yponomeutini, equivalent to Yponomeutidae sensu Friese [20] and Yponomeutini sensu Moriuti [27]. The 8–27 gene degen1 result, being stronger and concordant with morphology, seems more persuasive than the nt123 placement for *Thecobathra*. We therefore provisionally recognize a subfamily Yponomeutinae composed of Niphonymphini+Yponomeutini.

Our analyses provide robust, consistent evidence on the initial divergences within Yponomeutini as sampled here. *Metanomeuta* branches off first (Fig. 2, **node 21**; BP = 100, nt123), followed by *Paraswammerdamia* (Fig. 2, **node 22**; BP = 99, nt123). *Yponomeuta* is strongly paired with *Teinoptila* (Fig. 2, **node 23**; BP≥94, all analyses), and relationships among the four sampled species of *Yponomeuta* (Fig. 2, **nodes 24, 25, 26**) are also very strongly resolved. The remaining Yponomeutini comprise an assemblage whose monophyly is weakly supported by nt123 (Fig. 2, **node 28**; BP = 56, nt123) and weakly contradicted by degen1, which allies *Klausius* instead with *Teinoptila*+*Yponomeuta* (BP = 57, tree not shown). The remainder of the assemblage (Fig. 2, **node 28**) divides into two strongly supported clades, one consisting of *Cedestis*+*Zelleria retiniella* (Fig. 2, **node 29**; BP = 100, nt123), and the other (Fig. 2, **node 32**; BP = 90, nt123) containing additional species of *Zelleria* plus three other genera, relationships among which are not clearly resolved. These results strongly contradict all previous hypotheses about relationships within Yponomeutini, including Kloet & Hincks [93], Moriuti [27], Heppner [1] and Ulenberg [86]. In addition, our data provide strong evidence for polyphyly of *Zelleria* (Fig. 2, **nodes 30, 34**). Clearly there is much further work to be done on the systematics of Yponomeutini.

### Relationships within the YPG Clade

In our analyses, the sister group to Yponomeutidae consists of Ypsolophidae, Plutellidae and Glyphipterigidae. Grouping of the latter three families, the ‘YPG clade’, is very strongly supported (Fig. 2, **node 36**; BP = 98–100, all analyses). This clade has never been proposed previously, and no morphological synapomorphies are known. The basal split within the YPG clade, also very strongly supported, unites Plutellidae and Glyphipterigidae to the exclusion of Ypsolophidae (Fig. 2, **node 45**; BP≥99, all analyses).

Monophyly of Ypsolophidae including *Ochsenheimeria* is very strongly supported by our data (Fig. 2, **node 37**; BP = 100, all analyses). A similar result was reported by Mutanen et al. [29]. The enigmatic *Ochsenheimeria* group was long assigned to Tineoidea before Kyrki [25] allied it with Yponomeutoidea. Kyrki [26] proposed eight synapomorphies for Ypsolophidae including Ochsenheimeriinae: hindwing veins with Rs and M_1_ stalked or coincident; male genitalia with tegumen deeply bilobed at the anterior margin; tuba analis membranous and densely setose; phallus with two cornuti or cornutal zones; female genitalia with long anterior and posterior apophyses; termination of ductus seminalis on ductus bursae close to ostium; signum elongate, band-like, usually with two transverse ridges; and, pupal cremaster without setae. Heppner’s [1] placement of Ochsenheimeriinae (raised to the family level) as sister group to all other yponomeutoids (Fig. 1B) is strongly rejected by our data. Our data likewise reject proposals by Moriuti [27] and Heppner [1] to merge Ypsolophidae minus Ochsenheimeriinae into Plutellidae.

Within Ypsolophidae sensu Kyrki, our data provide somewhat contradictory evidence on the basal split. In all analyses that include synonymous change, Ypsolophinae are monophyletic, excluding *Ochsenheimeria*, with very strong support (Fig. 2, **node 38**; BP = 100, nt123). In contrast, under degen1, *Ochsenheimeria* is nested two nodes deep within Ypsolophinae, as sister group to *Bhadorcosma*, with 68% bootstrap support, contradicting two groupings (Fig. 2, **nodes 38, 39**) that have ≥99% bootstrap under nt123. While the signal from nt123 is stronger, we cannot confidently rule out the hypothesis of a paraphyletic Ypsolophinae [31] until this striking conflict is explained. Apart from the position of *Ochsenheimeria*, however, our data provide very strong resolution of all relationships within Ypsolophinae as sampled here (Fig. 2, **nodes 39, 40, 41**; BP = ≥99, nt123). *Ypsolopha* is always paraphyletic in our trees, with respect to either *Bhadorcosma* and *Ochsenheimeria* (degen1) or *Bhadorcosma* alone (all other analyses). Ypsolophidae are a cosmopolitan group of 5 genera and160 described species, most diverse in the Palearctic [2]. The larvae of Ypsolophinae are most often leaf webbers on woody plants, of many different families, while those of Ochsenheimeriinae are leaf miners and borers in Poaceae, Cyperaceae and Juncaceae (Poales).

### Relationships within the PG Clade

A sister group relationship between Plutellidae and Glyphipterigidae, very strongly supported by our data (Fig. 2, **node 45**; BP≥99, all analyses), has not been previously proposed. Given the exceptionally robust molecular evidence, a search for morphological synapomorphies seems warranted. Two possible candidates, hypothesized by Kyrki ([26], but see [31]) to unite Plutellinae and Acrolepiinae (now part of Glyphipterigidae), are lamellae postvaginales of the female genitalia consisting of two setose lobes, and loosely meshed cocoons.

Our analyses provide strong and consistent support for monophyly of Plutellidae (Fig. 2, **node 42**; BP = 93, nt123). Like Mutanen et al. [29], we find that the so-called “mega-plutellids” of New Zealand and Tasmania, here represented by *Proditrix* and *Doxophyrtis*, are actually nested within Glyphipterigidae: Orthoteliinae, as sister group to *Orthotelia* (Fig. 2, **node 49**; BP = 100, all analyses). Within Plutellidae sensu stricto [2] as sampled here, our data strongly support a basal split between a North Temperate “core” group consisting of *Plutella* and allies (Fig. 2, **node 44**; BP = 100, all analyses), and a tropical lineage (Fig. 2, **node 43**; BP≥93, all analyses) here represented by the Namibian *Deryaxenistis* and an undescribed genus from Mexico. The plutellid association for *Deryaxenistis*, previously tentative [94], [95], is here strongly confirmed. We suspect that this tropical plutellid lineage is greatly under-explored. Its characterization will probably result in a new morphological definition for the family. Kyrki [25] characterized Plutellidae in the restricted sense (*Plutella*-group auct) by male genitalia with curved gnathal processes surrounding the anal tube. This feature, however, is not found in the tropical clade, which may deserve subfamily status. Plutellidae are a cosmopolitan group of 48 genera and150 described species, most diverse in the Australoceanian region [2]. The larvae are typically skeletonizing leaf webbers [31]. More than half of the host records are from Brassicales.

The monophyly of Glyphipterigidae is very strongly supported in all of our analyses (Fig. 2, **node 52**; BP = 98, nt123) except degen1 and the codon model. The conflict concerns a newly-discovered, strongly-supported Neotropical clade of probable Orthoteliinae (Fig. 2, **node 47**; BP = 96, nt123). Under degen1, this clade branches off at the base of the PG clade in the ML tree, but with very weak support; the bootstrap value is actually higher (49%) for glyphipterigid monophyly. Like Mutanen et al. [29], we find Glyphipterigidae to consist of three subfamilies, Glyphipteriginae, Acrolepiinae and Orthoteliinae. Previous hypotheses based on morphology have sometimes included both Glyphipteriginae and Orthoteliinae (Table 1), but never Acrolepiinae, which have been variously treated as a subfamily of Plutellidae [26] or as a family related to Lyonetiidae and Heliodinidae [1]. Morphological synapomorphies for Glyphipterigidae in the new sense [2] have yet to be discovered. Kyrki & Itämie [96] and Kyrki [26] proposed eight synapomorphies for Glyphipterigidae excluding Acrolepiinae. Three of these – antenna without a pecten, male genitalia without teguminal processes, and larva endophagous – are also common in Acrolepiinae. These traits are also widespread in other lepidopteran lineages, however, leaving their phylogenetic significance uncertain. Within Glyphipterigidae, our data very strongly group Acrolepiinae with Glyphipteriginae to the exclusion of Orthoteliinae (Fig. 2, **node 53**; BP≥95, all analyses). Mutanen et al. [29] reported a similar result.

Our analyses favor a broad concept of the formerly monobasic Orthoteliinae (Fig. 2, **node 46**) that includes both the New Zealand/Tasmanian “mega-plutellids” (Fig. 2, **node 50**), as proposed by Heppner [97] and corroborated also by Mutanen et al. [29], and an assemblage of undescribed genera and species from the Neotropical region. This definition of the subfamily is strongly supported (Fig. 2, **node 46**; 89≤BP≤93) by all analyses except degen1 and the codon model, which, as noted earlier, very weakly place a subclade of Neotropical species (Fig. 2, **node 47**) at the base of either Glyphipterigidae or the PG clade (BP<<50; trees not shown). No morphological synapomorphies are apparent for Orthoteliinae in the new sense.

Within Orthoteliinae, the “mega-plutellids” (Fig. 2, **node 50**) appear closely related to the monobasic Palearctic type genus *Orthotelia* (Fig. 2, **node 49**; BP = 100, all analyses), while the Neotropical fauna may prove to constitute the paraphyletic basal lineages of the subfamily. One undescribed genus from Chile (“CL67”) is strongly supported as the nearest relative to the core group that includes *Orthotelia* (Fig. 2, **node 48**; 81≤BP≤96, all analyses), while the remaining Neotropical exemplars form a strongly supported clade (Fig. 2, **node 47**; BP = 96, nt123) that is sister group to all other orthoteliines. Further exploration of the Neotropical biodiversity of Orthoteliinae is clearly desirable. Within the mega-plutellid group (Fig. 2, **node 50**), no analysis yielded strong support for monophyly of *Proditrix* (Fig. 2, **node 51**; BP≤52, all analyses), while degen1 grouped *Doxophyrtis*+*Proditrix nielseni* to the exclusion of *P. gahniae*, with 86% bootstrap (denoted by dotted arrow in Fig. 2). Thus, *Proditrix* may be paraphyletic with respect to *Doxophyrtis*. The Orthoteliinae as here delimited contain 6 genera and 14 described species. The species with known hostplants are typically borers within monocots (>90% of host records).

Monophyly for Acrolepiinae is very strongly supported by our data (Fig. 2, **node 40**; BP = 100, all analyses). Kyrki [25] proposed four synapomorphies for acrolepiines [31]: reduction of the tegumen, teguminal processes, and gnathos; basal widening of the phallus; stalking of hindwing veins M_1_+M_2_; and stalking of hindwing veins M_3_+CuA_1_. However, the first of these, involving reduction of the tegumen, is also common in Glyphipteriginae. In addition, stalking of M_3_+CuA_1_ is found in *Sericostola* (Glyphipteriginae), though not in other glyphipterigine genera for which wing venation is known. Among Acrolepiinae as sampled here, our data strongly favor the grouping of *Acrolepiopsis*+*Digitivalva* (Fig. 2, **node 55**; BP = 100, all analyses) to the exclusion of *Acrolepia* (Fig. 2, **node 56**; BP = 87–100, all analyses). Acrolepiinae are a cosmopolitan group of 4 genera and 87 described species, most diverse in the Palearctic. The larvae are internal feeders in leaves, stems, flower buds and seeds of herbaceous plants, either monocots (*Acrolepiopsis*) or asterids (*Digitivalva*, *Acrolepia*).

Our analyses very strongly support monophyly for Glyphipteriginae as sampled here (Fig. 2, **node 57**; BP = 100, all analyses). Kyrki & Itämie [96] proposed three possible synapomorphies for Glyphipteriginae [31]: a conical male 8^th^ abdominal segment with an enlarged tergum; a vestigial M-stem and CuP in the forewing venation; and approximation (not stalking) of hindwing veins M_3_ and CuA_1_. Dugdale et al. [31] note that adult diurnality and a characteristic rhythmic raising and lowering of the wings while at rest may be additional synapomorphies. All divergences within Glyphipteriginae as sampled here are strongly to very strongly supported by nt123 (Fig. 2, **nodes 57–62**; BP 80–100, nt123), and contradicted in only two instances, weakly, by degen1. In our tree, *Glyphipterix quadragintapunctata* is the sister group to a strongly supported clade comprising all remaining Glyphipteriginae including the four other *Glyphipterix* species sampled (Fig. 2, **node 58**; BP = 100, all analyses). The two other genera sampled, *Diploschizia* and *Lepidotarphius*, each have sister groups consisting nearly or entirely of subsets of *Glyphipterix* species, rendering *Glyphipterix* paraphyletic with respect to both. According to Dugdale et al. [31], about two thirds of the species of glyphipterigines are placed in the cosmopolitan type genus, while many of the 20+ other genera are monobasic. Thus, *Glyphipterix* might prove paraphyletic with respect to other genera as well. Glyphipteriginae are a cosmopolitan group of 25 genera and 397 described species, most diverse in the Australoceanian and Oriental regions. The larvae are typically endophagous in the leaves or stems of commelinid monocots.

### Host Plant Associations

Previous hypotheses about life history evolution and biogeography of Yponomeutoidea (e.g. [3], [4], [20], [27], [86]) have been few, and their evaluation has been hampered by the lack of a robust phylogeny. In this and the next section we review trends in these features in light of our molecular phylogeny, as summarized in Figures 6 and 7.

To characterize the evolution of larval host plant associations, we sought to assess the degree of conservatism with respect to the new ypnomeutoid phylogeny, of mode of feeding, diet breadth (diversity of plant taxa used by individual species), host plant growth form, and host plant taxon membership at the family level and above. We also sought to infer the ancestral conditions and evolutionary directionality of these traits, for Yponomeutoidea as a whole and for subgroups thereof.

Larval feeding mode in the broad sense of internal versus external feeding is strongly conserved at the subfamily level and family level in yponomeutoids (Figure 7). Of the 16 subfamily or family clades identified by our phylogeny, only two show substantial variation in this trait. In Heliodinidae, internal feeding is numerically dominant but several early branching are external feeders, possibly representing the ancestral habit [84]. In Yponomeutidae external feeding is nearly universal, whereas internal feeding, specifically mining in conifer needles, is restricted to several species of the derived genera *Zelleria* and *Cedestis* [20], [31]. Despite this stability at the family and subfamily level, however, transitions between internal and external feeding are frequent enough to obscure the deeper-level history of this trait within Yponomeutoidea. For example, parsimony optimization across the entire phylogeny is unable to assign an unambiguous state to any ancestor below the family level (Figure 7). In this frequency of transition between internal and external feeding, Yponomeutoidea contrast strikingly with their nearest relatives, the possibly paraphyletic Gracillarioidea, within which internal feeding is universal.

Although here scored as “external feeding”, Scythropiidae (monospecific), as well as some species of Praydidae, Yponomeutidae, Heliodinidae and possibly other families, actually show an intermediate condition, in which initially leaf-mining larvae subsequently switch to become external leaf webbers. Analogous ontogenetic shifts from internal to external feeding are seen in a number of non-ditrysian groups as well [3], and may represent a pathway by which external feeding arises over evolutionary time as well. External feeding in yponomeutoids, as in most other so-called microlepidopterans, is not fully equivalent to that seen in Macroheterocera (sensu [2]), in that the larvae are not fully exposed, but rather concealed in some way, e.g. by leaf webbing. Nonetheless, given the multiple evolutionary transitions between internal and external feeding now identified, Yponomeutoidea offer promising material for further studies of the causes and consequences of this fundamental feature of evolution in Lepidoptera and other holometabolous insect phytophages [98].

A second aspect of yponomeutoid larval host use that shows striking phylogenetic conservatism is diet breadth. Oligophagy, defined as using plants of a single order, appears to be nearly universal, characterizing >96% of the 448 yponomeutoid species for which we found host records. Moreover, nearly all oligophagous yponomeutoids use only one plant family. We may be under-estimating the incidence of polyphagy, defined as using two or more plant orders, because for many species only a single host record exists. On the other hand, it also is possible that some of the 14 species that have been recorded from two or more plant families represent undetected host-specific sibling species complexes. Whatever the exact incidence of polyphagy in Yponomeutoidea turns out to be, it clearly seems to be dramatically less than that reported for many groups of Apoditrysia, particularly in Macroheterocera [3], [99]. Nonetheless, yponomeutoids, like many other insect herbivore clades in which individual species are mostly oligophagous, collectively use an enormous range of host plant families (see below). It may be that models of diversification of insect herbivore species and host associations that depend on plasticity of host use (e.g. [100]) are less applicable to clades of oligophages such as yponomeutoids than to lepidopteran groups with greater mean diet breadth.

A third phylogenetically conserved aspect of yponomeutid host use is growth form of the host plant. Nearly all of the 16 subfamily/family clades supported by our molecular analyses feed on either woody or herbaceous plants, but not both (Fig. 6). The main exceptions are in Plutellidae and Yponomeutini. Most Plutellidae feed on Brassicales or other herbaceous taxa, but eight species of *Chrysorthenches* have been recorded from Podocarpaceae. Most Yponomeutini feed on woody plants, but about 20% feed on herbaceous Saxifragales. Parsimony optimization of herbaceous versus woody plant use on the molecular phylogeny (see Figure 6), when the nearest outgroups, Gracillarioidea, are included, reconstructs an ancestral association with woody plants, followed by relatively few independent origins of herb feeding, in Yponomeutini, the HSB clade and the YPG clade.

Finally, association with particular plant families, orders or more inclusive clades is conserved to a variable but always obviously non-random extent, within and sometimes between the 16 major yponomeutoid clades. There is some suggestion that host-taxon conservatism is stronger among herb feeders than among woody plant feeders, as previously reported for other lepidopterans [99], [101]. Most of the taxa with pronounced fidelity to single or closely-related plant families are herb feeders (Fig. 6). For example, Bedelliidae are nearly restricted to Convolvulaceae; Heliodinidae feed almost exclusively on Nyctaginaceae or other Caryophyllales; Ochsenheimeriinae are known only from Poales; and, the great majority of Glyphipteriginae feed on commelinid monocots.

Among woody-plant feeders, the only comparable example is Attevidae, which feed almost exclusively on Simaroubaceae. Larger woody-plant-feeding clades are typically spread across many plant families and orders, with several, most notably Argyresthiidae, Ypsolophinae and Yponomeutini, using conifers as well as angiosperms as hosts. The Lyonetiinae, for example are recorded from 17 plant families in 10 orders, belonging to major clades [65] including magnoliids, basal eudicot lineages, basal core eudicot lineages, rosids and asterids (Fig. 6). As with other woody-plant-feeding clades, they are most often associated with rosids, particularly Rosales and Fabales, orders that are especially characteristic of north temperate forests. A few woody-plant feeding clades or subclades thereof show unusually frequent association with particular plant clades. The most notable example is *Yponomeuta*, in which 29 of the 42 species with recorded hosts feed on Celastraceae. Several other genera of Yponomeutini also include species feeding on Celastraceae. Our phylogeny, in which the Celastraceae-restricted *Teinoptila* is strongly supported as the sister group to *Yponomeuta*, is consistent with the conclusion of Turner et al. [102] that Celastraceae is the ancestral host for *Yponomeuta*. However, Celastraceae are unlikely to be the ancestral hosts for Yponomeutidae as a whole (contra [86]), as neither Niphonymphini nor Saridoscelinae feed on this family.

### Biogeography

Yponomeutoidea have been conventionally considered to be a primarily North Temperate group that is most diverse in the Palearctic region. Tabulation of the zoogeographical composition of the 16 tribe, subfamily and family clades supported by our phylogeny (Figure 7) suggests that this view needs modification. It is indeed the case that in a majority of lineages, nine of 16, species diversity is highest in the Palearctic, equaling or exceeding 50% of total diversity in five of these. However, half of the lineages, eight of 16, are now known to be at least represented in all major zoogeographic regions. Four other yponomeutoid groups have more restricted distributions but are still widespread: Ypsolophinae are nearly absent from the Southern Hemisphere; Ochsenheimeriinae and Niphonymphini are restricted to the Old World; Attevidae are pantropical, extending into the Nearctic Region. Two groups show strongly disjunct distributions. In Saridoscelinae, one of the two genera occurs in the Palearctic and Oriental regions, whereas the other is restricted to the Nearctic and Neotropical regions. Orthoteliinae are found in the Australian region, in Europe, and as demonstrated here for the first time, in the Neotropical region. On-going taxonomic revisions in Ypsolophinae, Yponomeutini, and Argyresthiidae by the first author show that in these groups, Neotropical species diversity has been significantly underestimated. The same may hold true for tropical diversity of yponomeutoids in general.

## Summary and Conclusions

### Phylogeny and Classification

Our molecular results offer substantial clarification of yponomeutoid relationships at multiple levels of classification:

1 We find consistent support, rising to very strong (BP = 99%) when rogue taxa are removed, for monophyly of a concept of Yponomeutoidea close to that of Kyrki [25], [26].
2 With one exception, our data are consistent with recognition of all 10 yponomeutoid families included in the classification of van Nieukerken et al. [2], and strongly support monophyly for eight of the nine families for which multiple representatives were sampled. We also find strong support for recognition of an 11th family, Scythropiidae **stat. rev.**, which was previously subordinate within Yponomeutidae.

The chief remaining uncertainty about yponomeutoid family-level classification concerns the subfamily Cemiostominae of Lyonetiidae. Our sole cemiostomine, *Perileucoptera*, is grouped (albeit weakly) with Lyonetiinae in the four-gene nt123 analysis, but is excluded entirely from Yponomeutoidea in all other analyses, suggesting conflict among genes. Such conflict may also underlie the inability of our AU test to reject monophyly for *Perileucoptera*+Lyonetiinae for the full data set, and the identification of *Perileucoptera* as a rogue taxon by RogueNaRok. We leave Cemiostominae in Lyonetiidae until its position is clarified, by further taxon sampling and perhaps gene tree/species tree analysis.

3 There is strong support for tribal and/or subfamily divisions within the three largest families, and for inter-generic relationships within all families for which two or more genera were sampled (Fig. 2).
4 We present a new working hypothesis for relationships among yponomeutoid families (Fig. 2) in which 7 of 8 nodes have at least moderate support (BP≥70), and 4 of 8 have strong support (BP≥80), in one or more analyses. It differs markedly from, and fits our data decisively better than, all previous hypotheses.

Our proposed classification and phylogeny are summarized in the following phylogenetically indented list, in which each taxon is taken to be the sister group of all following taxa at the same level of indentation, provided there is no intervening taxon with lesser indentation. Asterisks denote levels of bootstrap support for our proposed supra-familial clades (*, **, *** = BP≥70, 80, 90, respectively, in at least one analysis).

Superfamily Yponomeutoidea.

‘YYPGAL Clade’*.

‘YYPG Clade’:

Family Yponomeutidae.

Subfamily Yponomeutinae.

Tribe Yponomeutini.

Tribe Niphonymphini.

Subfamily Saridoscelinae.

‘YPG Clade’***:

Family Ypsolophidae.

Subfamily Ypsolophinae.

Subfamily Ochsenheimeriinae.

‘PG Clade’***:

Family Plutellidae.

Family Glyphipterigidae.

Subfamily Orthoteliinae.

Subfamily Glyphipteriginae.

Subfamily Acrolepiinae.

‘AL Clade’*:

Family Argyresthiidae.

Family Lyonetiidae.

Subfamily Lyonetiinae.

Subfamily Cemiostominae.

‘PAHSB Clade’*:

‘PA Clade’**:

Family Attevidae.

Family Praydidae.

‘HSB Clade’***.

Family Heliodinidae.

Family Bedelliidae.

Family Scythropiidae **stat. rev.**

### Host Associations

Yponomeutoidea show notable conservatism on the new phylogeny with respect to four aspects of larval host plant use:

1. **Internal versus external feeding** is strongly conserved at the family level, varying notably only within Heliodinidae and, to a much lesser extent, Yponomeutidae. Parsimony optimization on the molecular phylogeny (Figure 7) points to an internal feeding as the ancestral yponomeutoid condition, with external feeders arising several times independently. This transition may typically pass through an intermediate stage seen in several extant groups, in which larvae mine leaves in the first instar and subsequently switch to external feeding, living in a communal web and skeletonizing leaves.
2. **Diet breadth** is remarkably conserved across yponomeutoids (Figure 7), with oligophagy, defined as using plants of a single order, characterizing 96% of all species with recorded hosts (albeit uncorrected for singleton records). Moreover, nearly all oligophagous yponomeutoids use only one plant family. It seems therefore possible that at least some of the 14 species that have been recorded from two or more plant families, whose rate of incidence is highest in Lyonetiinae (17%) and Orthoteliinae (20%), will prove to represent undetected host-specific sibling species complexes.
3. **Growth form of host plants used** is also markedly conserved: with a few exceptions, the 16 family-group taxa supported by our phylogeny feed on either woody plants or herbaceous plants, but not both (Fig. 6). Parsimony optimization of herbaceous versus woody plant use on the molecular phylogeny (Figure 6), when the nearest outgroups, Gracillarioidea, are included, reconstructs an ancestral association with woody plants, followed by several independent origins of herb feeding, in Yponomeutini, the HSB clade and the YPG clade.
4. **Taxonomic affinity of host plants used**, at the level of plant family, order or more inclusive clade is conserved to a variable but always notable extent within each of the 16 family-group yponomeutoid clades (Figure 7). Most of the clades that are restricted mainly to a single plant family or order are herb feeders; woody plant feeders appear to shift somewhat more readily among plant orders, albeit typically within the rosid plant clade.

Given these strong initial phylogenetic patterns, yponomeutoids appear to provide promising material for future more detailed studies of the evolution and evolutionary consequences of host plant use in early-diverging ditrysian Lepidoptera.

### Biogeography

Our tabulation of yponomeutoid distributions in light of the molecular phylogeny shows that Yponomeutoidea are considerably more diverse outside the Palearctic than has previously been appreciated. Half (8) of the 16 family-group clades supported here are now known to occur in all major zoogeographic regions. The known distribution is expanded most markedly by our findings for two groups: Plutellidae, in which the North Temperate “core” group is shown to have a tropical sister lineage; and, the formerly monobasic, exclusively Palearctic Orthoteliinae, which are shown to include both Australoceanic and Neotropical lineages. From these results, in conjunction with recent revisionary studies, it seems likely that tropical and southern continent biodiversity of Yponomeutoidea, particularly that of the Neotropical Region, has been heretofore considerably under-estimated.

## Supporting Information

Figure S1 **A spreadsheet showing the included species with annotations of their classification, collecting locality, host plant families, identification check with DNA barcodes, sequence data completeness (fraction of total target sequence actually obtained) and GenBank accession numbers.** The eight genes initially sampled are shown to the left of the 11–19 additional genes sampled for a subset of taxa. The genes sampled for the 4-gene nt123 analysis are shown in bold.
(XLS)

Figure S2 **The best maximum likelihood tree found in nt123 analysis of the 4-gene, 139-taxon data set.** The four genes are listed in Figure S1. The tree is rooted with *Tischeria ekebladella*. Bootstrap values, when >50%, are shown above branches.
(PDF)

Figure S3 **The best ML tree found for nt12 (only) analysis of the 8–27 gene, 139-taxon data set, rooted with** ***Tischeria ekebladella*** **.** Bootstrap values, when >50%, are shown above branches.
(PDF)

Figure S4 **The best ML tree for nt3 (only) analysis of the 8–27 gene, 139-taxon data set, rooted with** ***Tischeria ekebladella*** **.** Bootstrap values, when >50%, are shown above branches.
(PDF)

Figure S5 **The best ML cladogram from** **Figure 2** **, with bootstrap values for the initial 8 genes (nt123 analysis).** Values for 109fin, 205fin, 208fin, and 3007fin are shown above branch, in that order; values for ACC, CAD, DDC and enolase are shown below branches. ‘−’ = node not recovered in the ML tree for that analysis. ‘*’ = bootstrap value <50%. ‘NA’ = bootstrap value undefined because sequence was obtained for ≤1 taxon for that that gene in that clade. Bootstrap supports for groups with missing taxa are calculated from the remaining taxa.
(PDF)
