# Supplementary material for: A Molecular Phylogeny for Yponomeutoidea (Insecta, Lepidoptera, Ditrysia) and Its Implications for Classification, Biogeography and the Evolution of Host Plant Use
Source: PLoS One. 2013 Jan 31;8(1):e55066. doi: 10.1371/journal.pone.0055066 (PMC3561450; doi:10.1371/journal.pone.0055066)
Supplement: Figure S2 — The best maximum likelihood tree found in nt123 analysis of the 4-gene, 139-taxon data set. The four genes are listed in Figure S1. The tree is rooted with Tischeria ekebladella. Bootstrap values, when >50%, are shown above branches. (PDF) [file pone.0055066.s002.pdf]

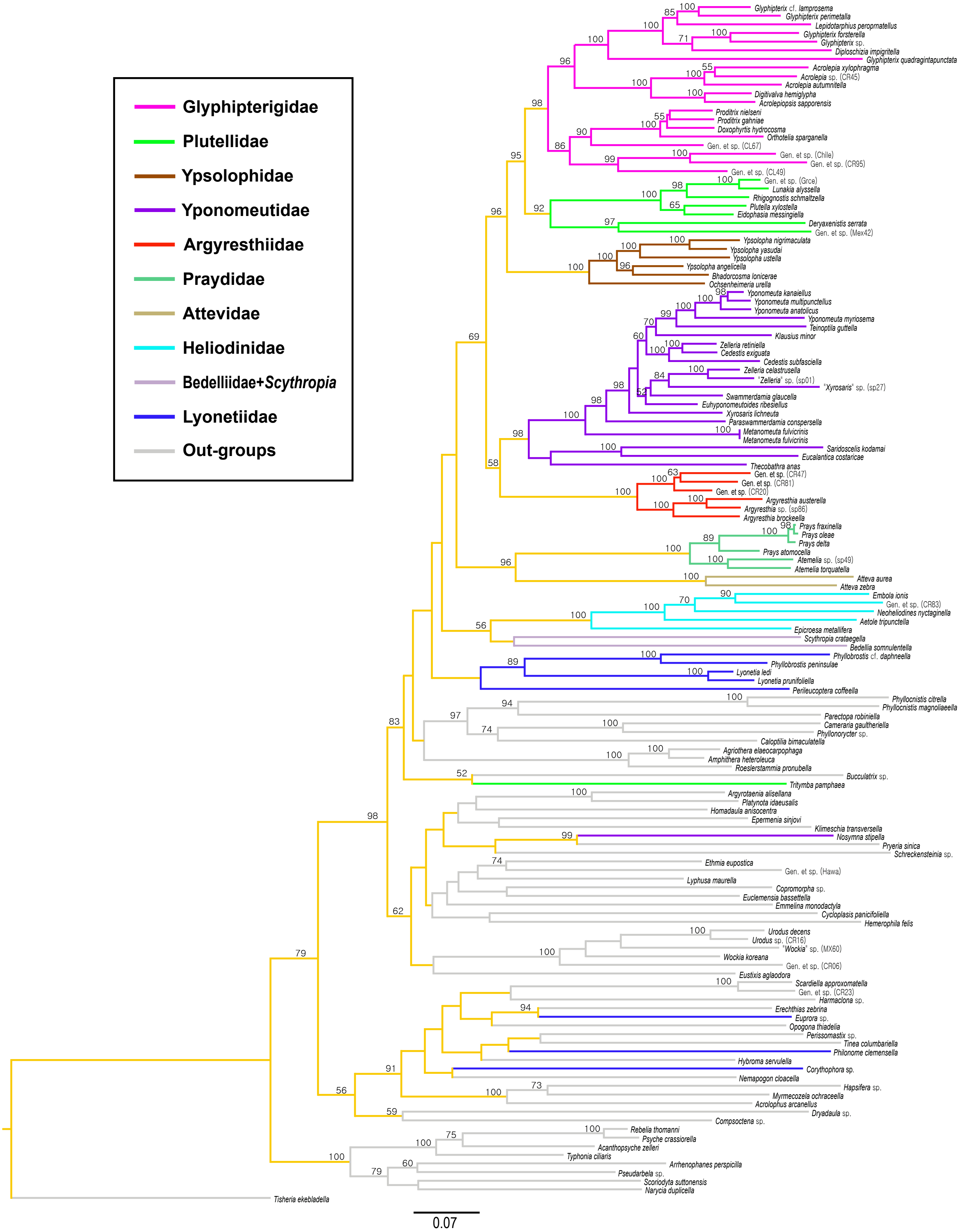

Glyphipterigidae
Plutellidae
Ypsolophidae
Yponomeutidae
Argyresthiidae
Praydidae
Attevidae
Heliodinidae
Bedelliidae+Scythropia
Lyonetiidae
Out-groups
Glyphipterix cf. lamprosema
Glyphipterix perimetalla
Lepidotarphius perornatellus
Glyphipterix forsterella
Glyphipterix sp.
Diploschizia impigritella
Glyphipterix quadragintapunctata
Acrolepia xylophragma
Acrolepia sp. (CR45)
Acrolepia autumnitella
Digitivalva hemiglypha
Acrolepiopsis sapporensis
Proditrix nielseni
Proditrix gahniae
Doxophyrtis hydrocosma
Orthotelia sparganella
Gen. et sp. (CL67)
Gen. et sp. (Chile)
Gen. et sp. (CR95)
Gen. et sp. (CL49)
Gen. et sp. (Grce)
Lunakia alyssella
Rhigognostis schmaltzella
Plutella xylostella
Eidophasia messingiella
Deryaxenistis serrata
Gen. et sp. (Mex42)
Ypsolopha nigrimaculata
Ypsolopha yasudai
Ypsolopha ustella
Ypsolopha angelicella
Bhadorcosma lonicerae
Ochsenheimeria urella
Yponomeuta kanaiellus
Yponomeuta multipunctellus
Yponomeuta anatolicus
Yponomeuta myriosema
Teinoptila guttella
Klausius minor
Zelleria retiniella
Cedestis exiguata
Cedestis subfasciella
Zelleria celastrusella
"Zelleria" sp. (sp01)
"Xyrosaris" sp. (sp27)
Swammerdamia glaucella
Euhyponomeutoides ribesiellus
Xyrosaris lichneuta
Paraswammerdamia conspersella
Metanomeuta fulvicrinis
Metanomeuta fulvicrinis
Saridoscelis kodamai
Eucalantica costaricae
Thecobathra anas
Gen. et sp. (CR47)
Gen. et sp. (CR81)
Gen. et sp. (CR20)
Argyresthia austerella
Argyresthia sp. (sp86)
Argyresthia brockeella
Prays fraxinella
Prays oleae
Prays delta
Prays atomocella
Atemelia sp. (sp49)
Atemelia torquatella
Atteva aurea
Atteva zebra
Embola ionis
Gen. et sp. (CR83)
Neoheliodines nyctaginella
Aetole tripunctella
Epicroesa metallifera
Scythropia crataegella
Bedellia somnulentella
Phyllobrostis cf. daphneella
Phyllobrostis peninsulae
Lyonetia ledi
Lyonetia prunifoliella
Perileucoptera coffeella
Phyllocnistis citrella
Phyllocnistis magnoliaeella
Parectopa robiniella
Cameraria gaultheriella
Phyllonorycter sp.
Caloptilia bimaculatella
Agriothera elaeocarpophaga
Amphithera heteroleuca
Roeslerstammia pronubella
Bucculatrix sp.
Tritymba pamphaea
Argyrotaenia alisellana
Platynota idaeusalis
Homadaula anisocentra
Epermenia sinjovi
Klimeschia transversella
Nosymna stipella
Pryeria sinica
Schreckensteinia sp.
Ethmia eupostica
Gen. et sp. (Hawa)
Lyphusa maurella
Copromorpha sp.
Euclemensia bassettella
Emmelina monodactyla
Cycloplasis panicifoliella
Hemerophila felis
Urodus decens
Urodus sp. (CR16)
"Wockia" sp. (MX60)
Wockia koreana
Gen. et sp. (CR06)
Eustixis aglaodora
Scardiella approxomatella
Gen. et sp. (CR23)
Harmaclona sp.
Erechthias zebrina
Euprora sp.
Opogona thiadelia
Perissomastix sp.
Tinea columbariella
Philonome clemensella
Hybroma servulella
Corythophora sp.
Nemapogon cloacella
Hapsifera sp.
Myrmecozela ochraceella
Acrolophus arcanellus
Dryadaula sp.
Compsoctena sp.
Rebelia thomanni
Psyche crassiorella
Acanthopsyche zelleri
Typhonia ciliaris
Arrhenophanes perspicilla
Pseudarbela sp.
Scoriodyta suttonensis
Narycia duplicella
Tisheria ekebladella
0.07
