# Supplementary material for: A Molecular Phylogeny for Yponomeutoidea (Insecta, Lepidoptera, Ditrysia) and Its Implications for Classification, Biogeography and the Evolution of Host Plant Use
Source: PLoS One. 2013 Jan 31;8(1):e55066. doi: 10.1371/journal.pone.0055066 (PMC3561450; doi:10.1371/journal.pone.0055066)
Supplement: Figure S3 — The best ML tree found for nt12 (only) analysis of the 8–27 gene, 139-taxon data set, rooted with Tischeria ekebladella . Bootstrap values, when >50%, are shown above branches. (PDF) [file pone.0055066.s003.pdf]

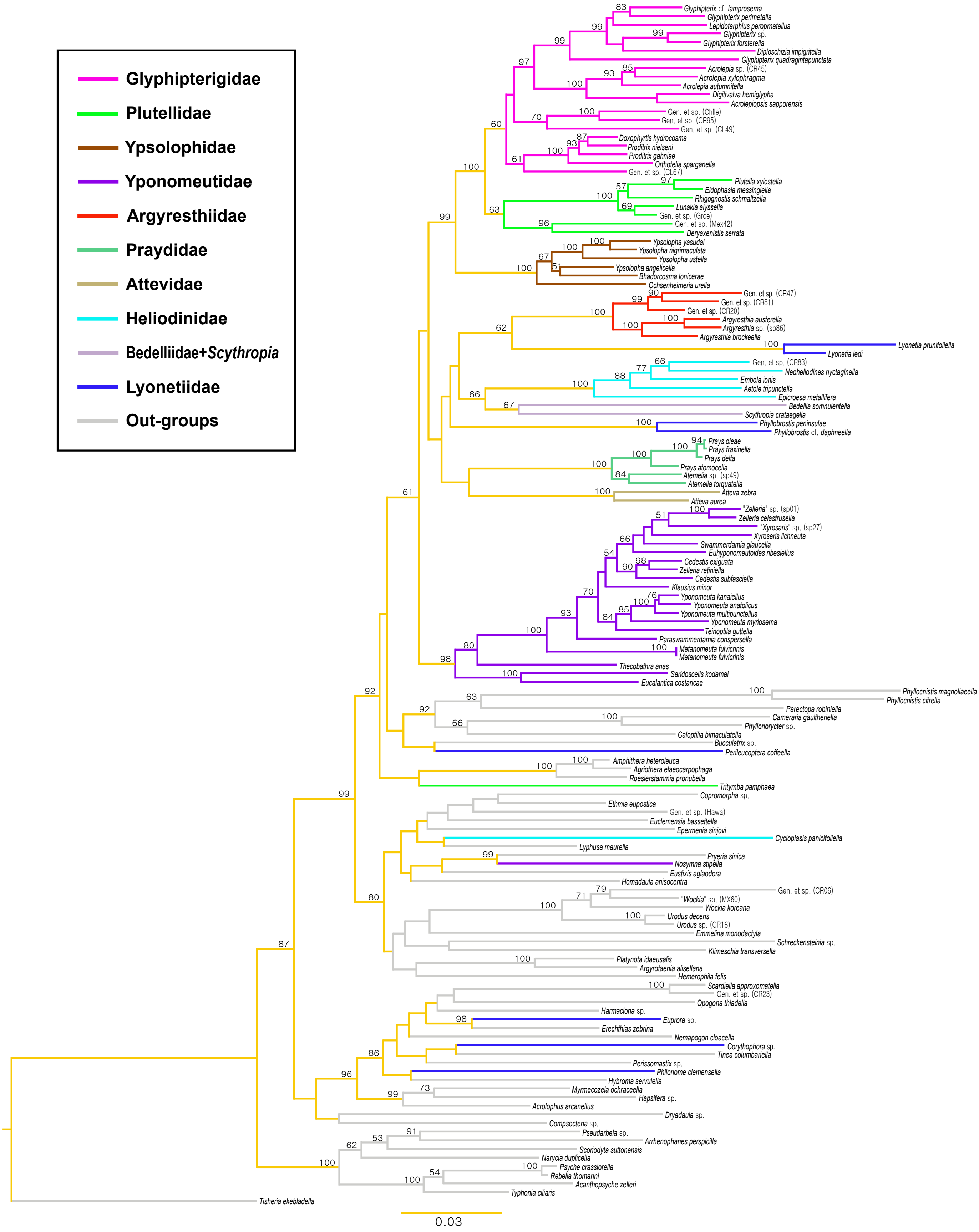
Glyphipterigidae
Plutellidae
Ypsolophidae
Yponomeutidae
Argyresthiidae
Praydidae
Attevidae
Heliodinidae
Bedelliidae+Scythropia
Lyonetiidae
Out-groups
Glyphipterix cf. lamprosema
Glyphipterix perimetalla
Lepidotarphius perornatellus
Glyphipterix sp.
Glyphipterix forsterella
Diploschizia impigritella
Glyphipterix quadragintapunctata
Acrolepia sp. (CR45)
Acrolepia xylophragma
Acrolepia autumnitella
Digitivalva hemiglypha
Acrolepiopsis sapporensis
Gen. et sp. (Chile)
Gen. et sp. (CR95)
Gen. et sp. (CL49)
Doxophyrtis hydrocosma
Proditrix nielseni
Proditrix gahniae
Orthotelia sparganella
Gen. et sp. (CL67)
Plutella xylostella
Eidophasia messingiella
Rhigognostis schmaltzella
Lunakia alyssella
Gen. et sp. (Grce)
Gen. et sp. (Mex42)
Deryaxenistis serrata
Ypsolopha yasudai
Ypsolopha nigrimaculata
Ypsolopha ustella
Ypsolopha angelicella
Bhadorcosma lonicerae
Ochsenheimeria urella
Gen. et sp. (CR47)
Gen. et sp. (CR81)
Gen. et sp. (CR20)
Argyresthia austerella
Argyresthia sp. (sp86)
Argyresthia brockeella
Lyonetia prunifoliella
Lyonetia ledi
Gen. et sp. (CR83)
Neoheliodines nyctaginella
Embola ionis
Aetole tripunctella
Epicroesa metallifera
Bedellia somnulentella
Scythropia crataegella
Phyllobrostis peninsulae
Phyllobrostis cf. daphneella
Prays oleae
Prays fraxinella
Prays delta
Prays atomocella
Atemelia sp. (sp49)
Atemelia torquatella
Atteva zebra
Atteva aurea
"Zelleria" sp. (sp01)
Zelleria celastrusella
"Xyrosaris" sp. (sp27)
Xyrosaris lichneuta
Swammerdamia glaucella
Euhyponomeutoides ribesiellus
Cedestis exiguata
Zelleria retiniella
Cedestis subfasciella
Klausius minor
Yponomeuta kanaiellus
Yponomeuta anatolicus
Yponomeuta multipunctellus
Yponomeuta myriosema
Teinoptila guttella
Paraswammerdamia conspersella
Metanomeuta fulvicrinis
Metanomeuta fulvicrinis
Thecobathra anas
Saridoscelis kodamai
Eucalantica costaricae
Phyllocnistis magnoliaeella
Phyllocnistis citrella
Parectopa robiniella
Cameraria gaultheriella
Phyllonorycter sp.
Caloptilia bimaculatella
Bucculatrix sp.
Perileucoptera coffeella
Amphithera heteroleuca
Agriothera elaeocarpophaga
Roeslerstammia pronubella
Tritymba pamphaea
Copromorpha sp.
Ethmia eupostica
Gen. et sp. (Hawa)
Euclemensia bassettella
Epermenia sinjovi
Cycloplasis panicifoliella
Lyphusa maurella
Pryeria sinica
Nosymna stipella
Eustixis aglaodora
Homadaula anisocentra
Gen. et sp. (CR06)
"Wockia" sp. (MX60)
Wockia koreana
Urodus decens
Urodus sp. (CR16)
Emmelina monodactyla
Schreckensteinia sp.
Klimeschia transversella
Platynota idaeusalis
Argyrotaenia alisellana
Hemerophila felis
Scardiella approximatella
Gen. et sp. (CR23)
Opogona thiadelia
Harmaclona sp.
Euprora sp.
Erechthias zebrina
Nemapogon cloacella
Corythophora sp.
Tinea columbariella
Perissomastix sp.
Philonome clemensella
Hybroma servulella
Myrmecozela ochraceella
Hapsifera sp.
Acrolophus arcanellus
Dryadaula sp.
Compsoctena sp.
Pseudarbela sp.
Arrhenophanes perspicilla
Scoriodyta suttonensis
Narycia duplicella
Psyche crassiorella
Rebelia thomanni
Acanthopsyche zelleri
Typhonia ciliaris
Tisheria ekebladella
0.03
