# Supplementary figures and images for: A Molecular Phylogeny for Yponomeutoidea (Insecta, Lepidoptera, Ditrysia) and Its Implications for Classification, Biogeography and the Evolution of Host Plant Use
Source: PLoS One. 2013 Jan 31;8(1):e55066. doi: 10.1371/journal.pone.0055066 (PMC3561450; doi:10.1371/journal.pone.0055066)

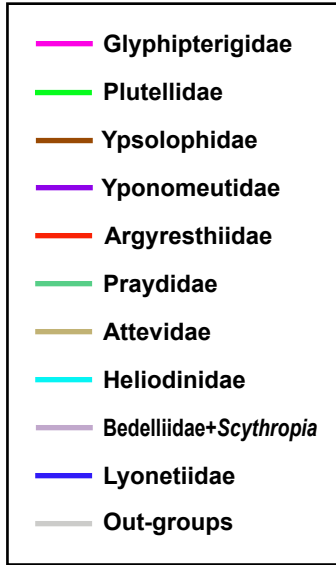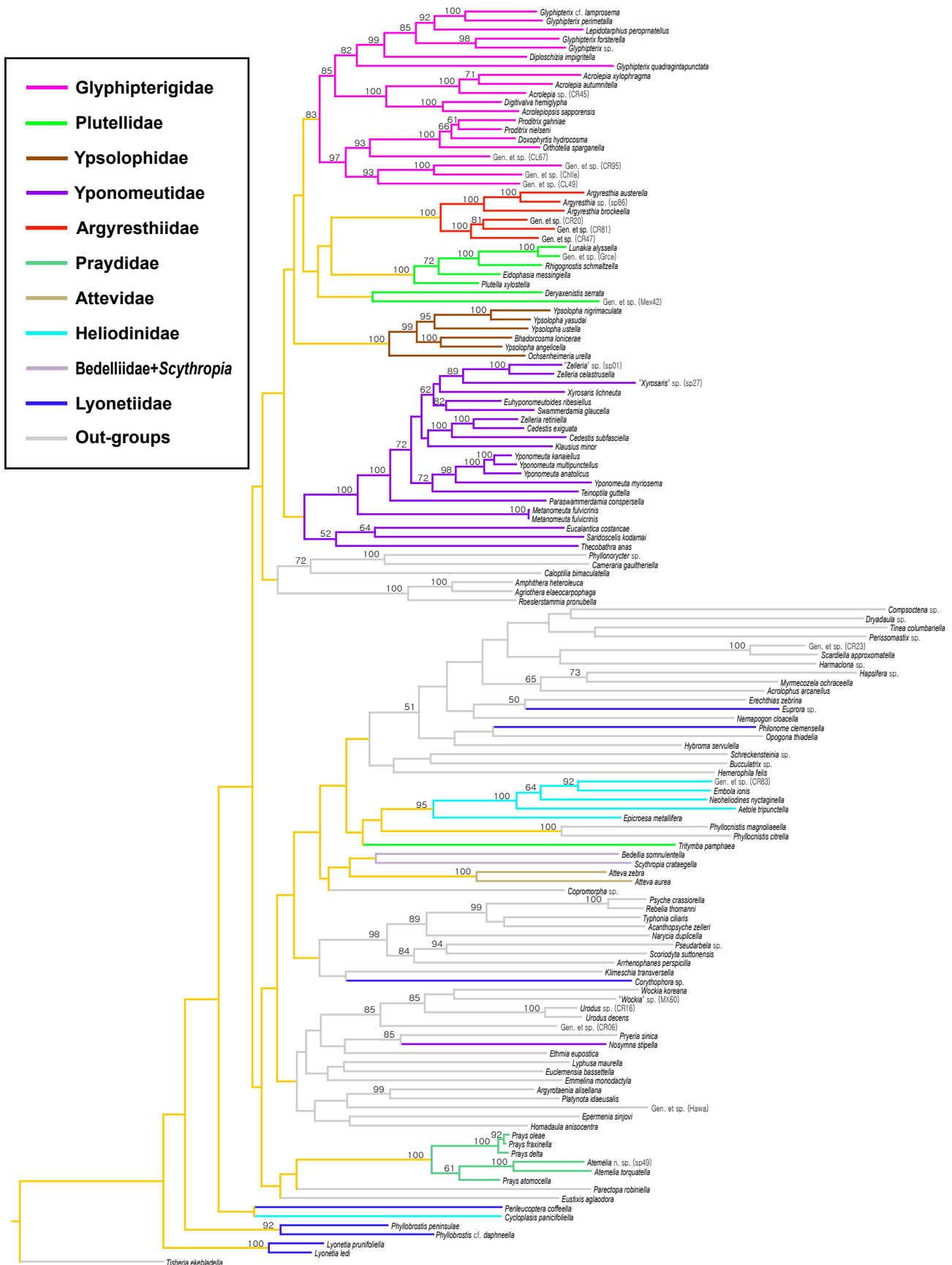

Supplement: Figure S4 — The best ML tree for nt3 (only) analysis of the 8–27 gene, 139-taxon data set, rooted with Tischeria ekebladella . Bootstrap values, when >50%, are shown above branches. (PDF) [file pone.0055066.s004.pdf]

109fin/265fin/268fin/3007fin  
ACC/CAD/DDC/enolase

**BP supports (%)**

- = unrecovered nodes  
\* = <50%

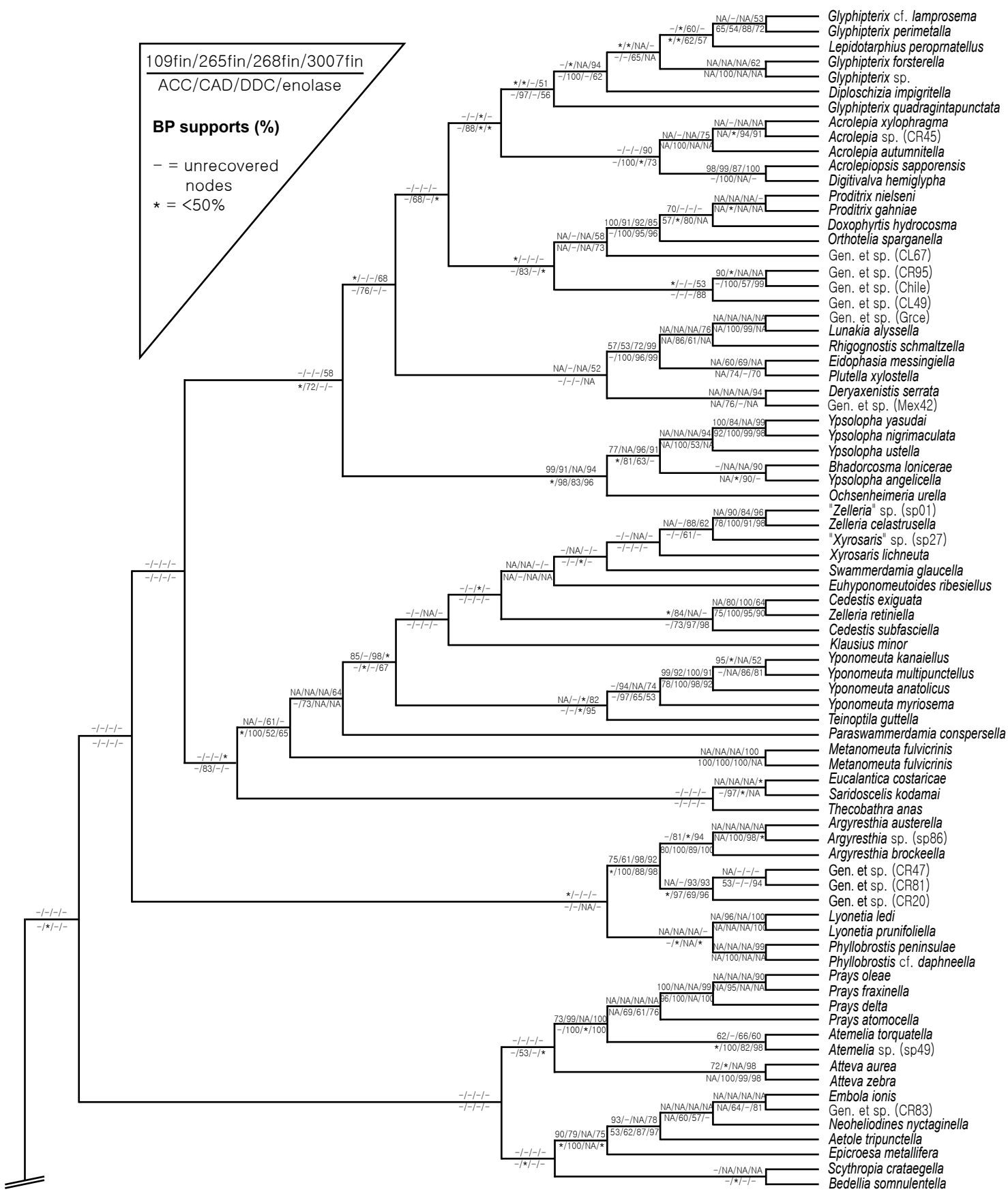

Supplement: Figure S5 — The best ML cladogram from Figure 2 , with bootstrap values for the initial 8 genes (nt123 analysis). Values for 109fin, 205fin, 208fin, and 3007fin are shown above branch, in that order; values for ACC, CAD, DDC and enolase are shown below branches. ‘−’ = node not recovered in the ML tree for that analysis. ‘*’ = bootstrap value <50%. ‘NA’ = bootstrap value undefined because sequence was obtained for ≤1 taxon for that that gene in that clade. Bootstrap supports for groups with missing taxa are calculated from the remaining taxa. (PDF) [file pone.0055066.s005.pdf]
